# Ubiquitin-proteasome system dysregulation in FAM111B-related poikiloderma and phenotypic spectrum expansion: new case reports and long-term follow-up

**DOI:** 10.1016/j.ebiom.2025.105864

**Published:** 2025-08-20

**Authors:** Virginie Vignard, Mike Maillasson, Anne Bigot, Sébastien Küry, Thomas Besnard, Martin Broly, Aurélie Guého, Emmanuelle Com, Erica Davis, Wallid Deb, Laëtitia Florenceau, Karen Sobriel, Grégoire Ménard, Betty Gardie, Alice Goldenberg, Joseph Porrmann, Randal Richardson, Léa Ruffier, Smail Hadj-Rabia, Stéphane Bézieau, Sébastien Barbarot, Frédéric Ebstein, Sandra Mercier

**Affiliations:** a Nantes Université, CNRS, INSERM, l’institut du Thorax, Nantes 44000, France; b Nantes Université, CNRS, INSERM, CRCI^2^NA, Nantes 44000, France; c Nantes Université, CHU Nantes, CNRS, INSERM, SFR Bonamy, Imp@ct Platform, Nantes 44000, France; d Sorbonne Université, Inserm, Institut de Myologie, Centre de Recherche en Myologie, Paris, France; e Nantes Université, CHU Nantes, Service de Génétique Médicale, Nantes 44000, France; f Univ Rennes, Inserm, EHESP, Irset (Institut de Recherche en Santé, Environnement et Travail) - UMR_S 1085, Rennes F-35000, France; g Univ Rennes, CNRS, Inserm, Biosit UAR 3480 US_S 018, Protim Core Facility, Rennes F-35000, France; h Center for Human Disease Modeling, Duke University Medical Center, Durham, NC 27705, USA; i Ecole Pratique des Hautes Etudes, EPHE, Université PSL, France; j Département de Génétique, CHU de Rouen, Rouen 76031, France; k Institute for Clinical Genetics, Universitätsklinikum, Technischen Universität Dresden, Dresden, Germany; l Department of Neurology, Gillette Children's Specialty Healthcare, St Paul, MN, USA; m Department of Dermatology and National Reference Center for Rare Skin Diseases and Genodermatoses (MAGEC), Hôpital Necker-Enfants Malades, AP-HP5, Paris, France; n INSERM U1163, Institut Imagine, Paris University, Paris, France; o Nantes Université, CHU Nantes, Service de Dermatologie, Nantes 44000, France

**Keywords:** Poikiloderma, Case report, FAM111B, Omics, Ubiquitin-proteasome system

## Abstract

**Background:**

Poikiloderma, hereditary fibrosing, with tendon contractures, myopathy, and pulmonary fibrosis (POIKTMP) is a rare genetic multisystemic fibrosing disorder caused by *FAM111B* gene mutations. Given its rarity, the molecular underpinnings of POIKTMP remain elusive. FAM111B, a trypsin-like serine protease, initially studied in cancer, exhibits germline variants not consistently linked to tumours, suggesting broader functions beyond cell proliferation.

**Methods:**

In this study, we compiled and compared the clinical features of 41 POIKTMP patients, which included the description of 4 newly identified cases. Functional studies involved the exploration of patient-derived cells carrying *FAM111B* missense variants using omics technologies.

**Findings:**

Our results show that the phenotypic spectrum of POIKTMP encompassed renal failure, dental anomalies, hypoparathyroidism, and potentially neuropathy. Notably, variants clustering within the D-box domain of FAM111B protein tend to present a more severe phenotype. Most importantly, loss of FAM111B expression perturbed ubiquitin-proteasome system (UPS) function, leading to increased content of ubiquitin-protein conjugates and a sterile type I interferon signature.

**Interpretation:**

These findings highlight a dysfunctional UPS as a potential central driver of POIKTMP's molecular pathogenesis, presenting promising therapeutic avenues.

**Funding:**

10.13039/100007393Association Française contre les Myopathies (AFM – 20760), Fondation Génavie (657298), Fondation Thellie, I-SITE NExT Junior Talent, Biogenouest, 10.13039/100015510Infrastructures en Biologie Santé et Agronomie (IBiSA) and 10.13039/501100004584Conseil Régional de Bretagne.

**Research in context**

**Evidence before this study**

Hereditary fibrosing poikiloderma with tendon contractures, myopathy, and pulmonary fibrosis (POIKTMP) is a rare multi-organ fibrosing disorder attributed to pathogenic variants in the *FAM111B* gene, yet its molecular pathogenesis remains poorly understood. Due to the scarcity of cases and limited access to biological samples, understanding disease pathogenesis remains a challenge. Nevertheless, unravelling this aspect is imperative for identifying reliable biomarkers and potential therapeutic targets.

**Added value of this study**

This study leverages omics technology to explore the proteomes of patients with POIKTMP. Our findings reveal an association between POIKTMP and proteasome dysfunction, as well as the emergence of a type I interferon (IFN) gene signature in patient-derived cells.

**Implications of all the available evidence**

The findings suggest that dysregulation of the ubiquitin-proteasome system (UPS) plays a role in disease pathogenesis. Both proteasome loss of function and type I IFN expression may serve as biomarkers for functional diagnostics and for classifying *FAM111B* variants of uncertain significance.

## Introduction

Poikiloderma is a heterogeneous group of rare multi-organ fibrosing disorders characterized by a distinctive set of dermatological signs combining erythema, telangiectasia, as well as hyperpigmentation and hypopigmentation, giving the skin a mottled appearance.^1^ Additionally, dermal atrophy is also quite prevalent. The underlying molecular mechanisms involved in the appearance of poikiloderma are not fully understood; however, they can be attributed to a wide range of factors, including several environmental triggers such as sun exposure, inflammation, oxidative stress, as well as genetic predisposition.^2^ Several monogenic forms of poikiloderma have been reported, with the majority of them being autosomal recessive in nature. These notably include Rothmund-Thomson syndrome (caused by biallelic variants in *ANAPC1*, RTS1 [MIM: 268400]; *RECQL4*, RTS2 [MIM: 618625]; *CRIPT*, RTS3 [MIM: 615789]; *DNA2*, RTS4 [MIM: 620819])^1^^,^^3^^,^^4^ Kindler syndrome (*FERMT1*, MIM: 173650),^5^ poikiloderma with neutropenia (*USB1*, MIM: 604173),^1^^,^^6^ or inflammatory poikiloderma with hair abnormalities and acral keratoses (*LTV1*, MIM: 620199).^7^ The only dominant form highlighted so far is the hereditary fibrosing poikiloderma with tendon contractures, myopathy and pulmonary fibrosis (POIKTMP), which is caused by monoallelic variants in *FAM111B* (MIM: 615704).^8^ To date, only 37 cases of POIKTMP have been reported, involving 21 unrelated families. The variants have arisen de novo in 16 sporadic cases and were inherited in an autosomal dominant manner in 5 families, including the South African family initially described in 2006.^9^ POIKTMP is often mistaken for RTS, primarily due to the similarity of their skin lesions, although the clinical spectrum of POIKTMP is broader. Indeed, despite the consistent hallmark of epidermal involvement resulting in poikiloderma, a feature present in all affected individuals, the POIKTMP phenotype exhibits significant variability among individuals.^10^ The first clinical sign is poikiloderma, which generally appears in the first six months of life and tends to remain stable over the long term. Hypotrichosis and hypohidrosis leading to heat intolerance, are usually present. Importantly, POIKTMP is also characterized by tendon contractures and progressive muscle weakness primarily resulting from adipose tissue infiltration into the muscle tissue. Other frequent associated signs include exocrine pancreatic insufficiency and liver damage.^10^^,^^11^ In adulthood, there is a predisposition to pancreatic adenocarcinoma, as well as a risk of life-threatening pulmonary fibrosis, leading to the replacement of normal lung tissue with fibrous tissue and scarring.^8^^,^^11^ As previously emphasized, all documented cases of POIKTMP described so far have arisen from dominant missense variants in the *FAM111B* gene, with the exception of a single family [p.(Lys421del)]. To date, eleven pathogenic missense variations have been identified, forming two clusters: the first cluster located upstream of the protein's catalytic site (cluster 1), and the second cluster situated within the trypsin-like serine protease domain (cluster 2). While establishing a genotype-phenotype correlation remains challenging, it has been suggested that the variants within cluster 2 might be associated with a more severe phenotype of the disease.^12^

The protein encoded by *FAM111B* belongs to the FAM111 family of serine proteases, which consists of only two members: FAM111B and its homologue FAM111A.^13^ Structurally, FAM111B shares 46% of homology with FAM111A, with the most homologous sequences corresponding to their catalytic domains and their two ubiquitin-like domains UBL1 and UBL2, whose specific functions have not yet been determined.^13^ Among the two proteases, the structure of FAM111A is more accurately characterized. It comprises three functional domains that confer specific roles in DNA replication: a trypsin-like catalytic domain, a PIP box (PCNA Interaction Protein) responsible for binding to the PCNA (Proliferating Cell Nuclear Antigen) protein, and a DNA binding domain.^14^ It has been recently proposed that FAM111A protects the DNA replication fork by removing DNA-protein crosslinks.^15^ Functional homology between FAM111A and FAM111B has been reported,^16^ however *FAM111A* dominant missense variants give rise to very different clinical phenotypes without dermatological involvement, such as Kenny-Caffey syndrome type 2 (MIM: 127000) and Gracile bone dysplasia (MIM: 602361).

The precise function of FAM111B is not fully understood, although its increased expression in tumours suggests a role in cell cycle regulation.^15^^,^^17^^,^^18^ However, the exact influence of FAM111B on cell proliferation remains contentious. Some studies indicate that FAM111B exhibits anti-apoptotic properties by degrading the cell cycle inhibitor p16 and facilitating the stabilization of the pro-apoptotic protein Bcl2.^17^^,^^18^ Conversely, other investigations propose a divergent role for FAM111B as a tumour suppressor. In this context, gain-of-function variants were observed to enhance protease activity, thereby elevating apoptosis.^19^ The oncogenic nature of FAM111B is additionally supported by the fact that its gene expression is suppressed by p53.^18^^,^^20^

Altogether, the physiological function of FAM111B remains unclear, making it difficult for healthcare professionals to inform the patients about the evolution, prognosis, and potential therapeutic levers relevant in POIKTMP. The objectives of our study were two fold: (i) firstly, to gain a better understanding of the natural history of the disease and search for genotype-phenotype correlations; and (ii) secondly, to elucidate the role of the FAM111B protein. To achieve this, we used immortalized fibroblasts from affected individuals as a cellular model. Employing a multi-omics strategy, we aimed to explore the functional consequences of *FAM111B* variants and knockdown on cellular processes.

## Methods

### Sex as a biological variable

Our study examined males and females, and similar findings were reported for both sexes. However, sex was not considered as a stratifying factor during the study design, as the selection criteria were solely based on the presence of POIKTMP-associated variants in the *FAM111B* gene, regardless of sex.

### Individual recruitment

Four new individuals from two independent families (Families A and B) were referred by their physicians (geneticists and neurologists) after *FAM111B* variant identification by whole exome sequencing in the two index cases and Sanger targeting sequencing in the mother (Table 1). In Family B, the father’s DNA was not available for molecular confirmation, and POIKTMP diagnosis was made based on phenotype. The clinical data of five individuals previously reported by our team have been updated, as they are being followed at our university hospital in Nantes, France, or in collaboration with French clinicians. An extensive review of the literature was carried out on the 32 other individuals already reported. Main clinical and molecular data are summarized in table (Supplementary Table S2).

**Table 1:** Clinical features of the POIKTMP subjects with FAM111B variants.

Case reports	Family A		Family B		Individuals with long-term follow-up^a^^,^^8^					Literature	Total (%)
Sex (individual)	M (I1)	F (I2)	F (I3)	M (I4)	M (F1)	M (F2)	F (F4)	F (F5)	M (F8)	15 F/17 M	n = 41 19 F/22 M
Age at last examination	11 y	47 y	35 y	48 y^b^	18 y	12 y	15 y	12 y	17 y	26.7 y	**26.1 y**
FAM111B variant (NM_198947.4)	c.1301A>C p.(Tyr434Ser)		c.1880G>C p.(Arg627Gly)	n/a	c.1879A>G p.(Arg627Gly)	c.1879A>G p.(Arg627Gly)	c.1883G>A p.(Ser628Asn)	c.1883G>A p.(Ser628Asn)	c.1874C>A p.(Thr625Asn)	11 variants	**13 variants**
Inheritance	inherited	de novo	assumed inherited	n/a	de novo	paternal	assumed de novo	de novo	de novo	12 de novo 13 inherited	**17 dn (50%)** **17 inh. (50%)**
Poikiloderma	+	+	+	n/a	+	+	+	+	+	32/32	**40/40 (100%)**
Hypotrichosis	–	+	+	n/a	+	+	+	+	+	27/30	**34/38 (89%)**
Hypohidrosis	+	+	+	n/a	+	+	+	+	–	24/27	**31/35 (89%)**
Muscle histological, biological or EMNG finding	n/a	n/a	n/a	n/a	+	n/a	+	+	**n/a**	9/11	**14/17 (82%)**
Tendon contractures	–	+	+	n/a	+	+	+	+	+	13/18	**20/26 (77%)**
Muscle impairment	–	+	+	n/a	+	+	+	+	+	16/23	**23/31 (74%)**
Other tegument abnormalities	+	+	+	n/a	+	–	+	+	+	19/29	**26/38 (68%)**
Muscle weakness	–	+	+	n/a	+	+	+	+	+	10/18	**17/26 (65%)**
Endocrine impairment	–	–	–	n/a	–	n/a	n/a	+	n/a	3/3	**4/8 (50%)**
Pancreas impairment	+	+	+	+	+	+	–	–	–	14/32	**20/41 (49%)**
Liver Impairment	+	–	–	n/a	–	+	–	–	–	16/32	**18/40 (45%)**
Growth retardation or hypotrophy	–	–	–	n/a	+	–	+	+	–	7/14	**10/22 (45%)**
Lung impairment	n/a	n/a	–	n/a	+	–	+	–	+	9/27	**12/32 (38%)**
Renal insufficiency	–	–	n/a	n/a	–	–	n/a	+	–	2/2	**3/8 (38%)**
Spinal deformation	–	+	+	n/a	+	–	–	–	–	3/12	**6/20 (30%)**
Abnormal blood count	–	–	–	n/a	+	–	+	–	–	4/12	**6/20 (30%)**
Teeth abnormalities	–	–	–	n/a	–	n/a	n/a	+	n/a	1/3	**2/8 (25%)**
Severe muscle impairment	–	–	–	n/a	+	–	+	+	–	2/32	**5/40 (13%)**
Ophthalmologic abnormalities	–	–	–	n/a	–	–	–	–	–	2/11	**2/19 (11%)**

n/a: not assessed.

a Already reported patients.^8^^,^^32^

b Deceased.

### Ethics

The study has been ethically approved by the CHU de Nantes-ethics committee (number CCTIRS: 14.556) and by CPP Ouest V (File 06/15) on 04/08/2015 (Ref MESR DC 2017 2987). Written informed consent was obtained from all participants for the disclosure of clinical data, the use of biological material, and the publication of photographs.

### Human samples

Human fibroblasts expressing wild-type *FAM111B* were isolated from sex- and age-matched healthy donors (controls) and fibroblasts carrying the p.(Arg627Gly) or p.(Ser628Asn) heterozygous *FAM111B* missense variants were derived from patients with POIKTMP with informed consent. Fibroblasts were maintained in culture in Dulbecco's Modified Eagle Medium (DMEM) 1X containing 1 g/L glucose, 5 mM glutamine and supplemented with 10% FCS (Eurobio). In some experiments, fibroblasts were exposed to 50 μg/mL cycloheximide (CHX, Merck) up to 24 h.

Peripheral blood mononuclear cells (PBMC) were isolated from blood draws from healthy individuals (controls) and POIKTMP patients with p.(Arg627Gly) or p.(Ser628Asn) heterozygous *FAM111B* missense variants. In summary, peripheral blood mononuclear cells (PBMCs) were isolated through Ficoll gradient centrifugation (Eurobio), subjected to three washes with PBS, and subsequently cryopreserved in FBS containing 10% DMSO before being stored in liquid nitrogen for future utilization. Isolated PBMCs were cultured and expanded in U-bottom 96-well plates, along with feeder cells. The culture medium used was RPMI 1640 supplemented with 10% human AB serum (Dutscher), and the expansion process included the addition of 150 U/mL IL-2 (Miltenyi Biotec) and 1 μg/μL L-PHA (Sigma). This expansion protocol followed the methodology outlined in the procedure by Fonteneau et al.^21^ Following a culture period of 3–4 weeks, the resting T cells were subjected to a washing step and subsequently frozen as dry pellets for subsequent investigations. HeLa cells (ATCC) were cultivated in Dulbecco's Modified Eagle Medium (DMEM) 1X containing 4.5 g/L glucose, 5 mM glutamine and supplemented with 10% FCS (Eurobio). In some experiments, HeLa cells were subjected to a 12-h treatment with 200 nM bortezomib (Selleckchem) or 200 nM bafilomycin A1 (InvivoGen).

### RNA-sequencing

Total RNA was extracted from fibroblasts, quality-checked using a 2100 Bioanalyzer (Agilent) and Qubit 2.0 (ThermoFisher), and only samples with RIN > 7 were processed. RNA-seq libraries were prepared with the KAPA Stranded mRNA-Seq Kit (Roche) following standard procedures, including mRNA capture, fragmentation, reverse transcription, adaptor ligation, and strand-specific amplification. Libraries were indexed, quality-checked, pooled in equimolar ratios, and sequenced (50 bp, single-end reads) on an Illumina HiSeq 4000 platform, yielding ∼66 million reads per sample. Sequencing data were demultiplexed with Bcl2Fastq, trimmed with TrimGalore/Cutadapt, and aligned to the GRCh37v75 human genome using STAR. Only uniquely mapped reads were retained. Gene counts were generated with HTSeq, and differential expression analysis was performed with DESeq2 in R. Genes with ≥10 reads were included. Gene set enrichment analysis was conducted to identify pathways associated with differential expression. Full details are provided in the Supplementary material.

### Partner identification by SPR-MS

Surface plasmon resonance (SPR) experiments were conducted using a Biacore™ T200 system (GE Healthcare) with CM5 chips and HBS-N buffer at 37 °C and 5 μL/min flow rate. A monoclonal anti-FAM111B antibody was immobilized via standard amine coupling (∼10,000 RU). Fresh RIPA lysates from control and patient-derived fibroblasts expressing wild-type or mutant FAM111B (p.Arg627Gly or p.Ser628Asn) were diluted and injected over the chip. Bound molecules were recovered by a micro-recovery method using 1 μL of 30 mM NaOH, isolated by air bubbles (sandwich elution). Each lysate was subjected to 30 recovery cycles to obtain sufficient material. Eluates were collected in Protein LoBind tubes (Eppendorf), frozen, and analysed by nano LC-MS/MS, followed by differential statistical analysis. Full protocol details are available in the Supplementary material.

### Nano LC-MS/MS analyses

Proteins from fibroblasts were extracted in 30 mM Tris buffer (pH 7.4) with 8 M urea, 4% CHAPS, and protease inhibitors by sonication and centrifugation, as previously described.^22^ SPR recovery samples were precipitated with acetone, centrifuged, and resuspended in UTH-PMax buffer for enzymatic digestion. Both SPR recovery and 10 μg of fibroblast lysates (control and mutant) underwent digestion as described.^23^ Samples were reduced with DTT, alkylated with iodoacetamide, and digested with a Trypsin/Lys-C mix. Digestion continued overnight, and peptides were purified using Phoenix cartridges. Approximately 300 ng of purified peptides were analysed by nanoLC-MS/MS with a nanoElute coupled to a TimsTOF Pro mass spectrometer (Bruker) in PASEF mode.^24^ Peptide and protein identification was performed using the Mascot search engine, comparing spectra to the UniProtKB human proteome database (release 03/2022; 20577 sequences) and a common contaminant database. A false discovery rate (FDR) was calculated, and peptide validation was performed using a Mascot score ≥30. Weighted spectral counts for identified proteins were calculated using Abacus,^25^ and analysed for significant differences using a beta-binomial test in Proline Studio.^26^

### Functional analysis of differentially expressed genes and proteins

Differentially abundant proteins (DAPs) were analysed using the Metascape online tool.^27^ Pairwise comparisons between stages (t-test p-value ≤ 0.05 and fold-change ≥ 2) were further analysed using Proteomaps and STRING.^28^^,^^29^ Proteomaps graphics were created using DAP gene lists and their normalized weighted spectra values, organized by KEGG pathways. Proteomaps were visualized with Voronoi diagrams, grouping proteins by similar functions.

#### Gene set enrichment analysis (GSEA)

A custom GSEA program was used to identify differentially regulated biological pathways in human fibroblasts expressing wild-type or mutant FAM111B, based on RNA-seq and MS data.

#### Clustering proteomics

Proteins were clustered using keywords from Uniprot across nine categories, and KMeans clustering was performed in RStudio. Principal Component Analysis (PCA) was used to visualize the clusters.

#### Protein-protein interaction (PPI) networks

PPI networks were generated using the STRING database with a minimum interaction score of 0.7. Node embedding techniques (node2vec, DeepWalk) were applied to reduce dimensionality, and Gene Ontology annotations were incorporated to create vector representations. This approach helped identify proteins related to FAM111B and predicted functionally similar proteins.

### Plasmid constructions and transfection

The cDNA sequence encoding wild-type *FAM111B* was amplified by RT-PCR from total RNA isolated from fibroblasts derived from a healthy individual. The amplified product was cloned into a custom-made N-terminal HA-tagged vector derived from pcDNA3.1/Zeo(+) (Invitrogen) using the KpnI and XhoI restriction sites. The c.1879A>G p.(Arg627Gly) *FAM111B* variant was generated via site-directed mutagenesis. All constructs were checked by Sanger sequencing using T7 promoter and BGH reverse primers (Eurofins). HeLa cells were transfected with plasmids using the jetPRIME® transfection reagent (Sartorius) following the manufacturer's instructions.

### SDS-PAGE and Western-blot analysis

Cell pellets were lysed using equal volumes of standard RIPA buffer (50 mM Tris pH 7.5, 150 mM NaCl, 2 mM EDTA, 1 mM N- ethylmaleimide, 10 μM MG-132, 1% NP40, 0.1% SDS) before undergoing protein quantification through a standard bicinchoninic acid assay (BCA) from Thermo Fisher Scientific. Typically, twenty to forty micrograms of proteins were resolved via 4–12% SDS-PAGE (70 V for 3 h) followed by transfer (15 V for 5 min) onto nitrocellulose or PVDF membranes. Following a blocking step using 3% BSA at room temperature, the membranes were subjected to incubation with specific primary antibodies overnight at 4 °C with gentle agitation. Primary antibodies (Supplementary Table S1) used in this study were directed against FAM111B (product number HPA038637, Atlas Antibodies), K48-linked ubiquitin-modified proteins (clone D9D5, Cell SignallingTechnology, Inc), β-actin (clone C4, Santa Cruz Biotechnology), α6 (clone MCP106, Enzo Life Sciences), PA28α (K232/1, laboratory stock), PA200 (product number NBP2-32575, Novus Biologicals), LC3B (product number 2775, Cell Signalling Technology, Inc) as well as HA (clone HA.11, BioLegend). After the primary antibody incubation, the membranes underwent three washes with TTBS/0.2% Tween-20, followed by 1-h incubation with secondary antibodies conjugated with HRP (anti-mouse or anti-rabbit, products numbers 7076S and 7074S, Cell Signalling Technology, Inc). Subsequently, proteins were visualized using an enhanced chemiluminescence detection kit (ECL).

### Native PAGE and proteasome in-gel peptidase activity assay

Cell pellets were lysed using ice-cold homogenization TSDG buffer (10 mM Tris pH 7.0, 10 mM NaCl, 25 mM KCl, 1.1 mM 232 MgCl_2_, 0.1 mM EDTA, 2 mM DTT, 2 mM ATP, 1 mM NaN_3_, 20% Glycerol) and protein extraction was achieved through freeze/thaw cycles in liquid nitrogen. The quantification of proteins in the soluble lysates was determined through the Bradford assay. Twenty micrograms of whole-cell lysates were separated on 3–12% gradient Bis-Tris gels (Thermo Fisher Scientific) starting at 150 V for 1 h and then transitioning to 250 V for 30 min, using an appropriate electrophoresis buffer (BN2011BX10, Invitrogen by Thermo Fisher Scientific). After the separation, the chymotrypsin-like activity of proteasomes was assessed by incubating the gels with 0.1 mM of the suc-LLVY-AMC fluorogenic peptide (Bachem) at 37 °C for 20 min in an overlay buffer consisting of 20 mM Tris, 5 mM MgCl_2_, pH 7,0. Subsequently, the proteasome bands were visualized by exposing the gel to UV light at 360 nm and were detected at 460 nm using an Imager (GelDoc, Biorad).

### HA immunoprecipitation

HeLa cells transfected with an HA-tagged version of either wild-type or p.(Arg627Gly) FAM111B were subjected to protein extraction using the lysis buffer provided in the μMACS™ HA Isolation Kit (Miltenyi Biotec). The lysis buffer was supplemented with 10 mM N-Ethylmaleimide (Merck), 10 μM MG-132 (Merck), and 1 × cOmplete™ EDTA-free Protease Inhibitor Cocktail (Roche). A total of 2 mg of protein from cell lysates was used for the immunoprecipitation of HA-FAM111B, performed according to the manufacturer’s instructions for the μMACS™ HA Isolation Kit. Immunoprecipitated proteins were analysed by SDS-PAGE followed by Western blotting using standard protocols.

### RNA isolation, reverse transcription and PCR analysis

Total RNA was extracted from dry cell pellets using the Nucleospin RNA II kit from Macherey Nagel following the manufacturer’s instructions. For subsequent real-time PCR, 1 μg of the isolated total RNA was reverse transcribed using the M-MLV reverse transcriptase (ProtoScript II Reverse Transcriptase, NEB). Targets were amplified using TB Green Premix Ex Taq II (Takara) on a StepOnePlus™ Real-Time PCR System (Life Technologies) in duplicates with self-designed primers specific for FAM111B, RPL13A and PPIA as well as FAM-tagged TaqMan™ Gene Expression Assays obtained from Thermo Fisher (*IFI27, IFI44L, IFIT1, ISG15, RSAD2, IFI44, MX1*). The cycle threshold (Ct) values for target genes were transformed into values of relative expression using the relative quantification (RQ) method, specifically the 2ˆ(-ΔΔCt) formula. Expression levels of the target gene were calculated relative to the cycle threshold (Ct) values of the GAPDH, HPRT, PPIA and RPL13A control housekeeping genes.

### Statistics

Clinical data for the cohort of Individuals with a variant in cluster 1 of the *FAM111B* gene and those with a variant in cluster 2 were compared using Fisher's exact test. Pairwise comparisons from data generated by western-blotting and qPCR are typically presented as means ± SEM and analysed by unpaired t-test, following a normality assessment with the Shapiro–Wilk test, or Mann–Whitney U test between two groups. All charts and statistical analyses were generated using GraphPad Prism version 8. A p-value < 0.05 was considered significant. Proteomic data were analysed by a beta-binomial test on weighed spectral counts and a p-value was calculated for each Protein Set using the R package BetaBinomial 1.2 implemented in Proline Studio.^26^ For the KEGG pathway enrichment analysis of differentially expressed genes (DEGs), the false discovery rate (FDR) was used to account for multiple comparisons.

### Role of funders

The funders had no involvement in the study design, data collection, analysis, interpretation, or the writing and editing of the manuscript.

## Results

### Expanding the clinical spectrum of POIKTMP and the implications for tailoring patient management strategies

The clinical and molecular data of the 41 individuals, including 4 new reported individuals, 5 cases with long-term follow-up, and a review of the literature, are summarized in Table 1 and detailed in Supplementary Table S2. Here, we report 4 new cases from 2 different families, denoted as Families A and B. Family A had its origin in Germany, and the diagnosis was established at the age of 7 in a boy (I1) who inherited POIKTMP from his mother (I2). Family B originated from Germany, Ireland and Norway. The diagnosis was made at the age of 35 in a female individual (I3). She inherited POIKTMP from her father (I4), who died of pancreatic adenocarcinoma at the age of 48, a predisposition already described in POIKTMP.^11^^,^^31^

**Fig. 1:**
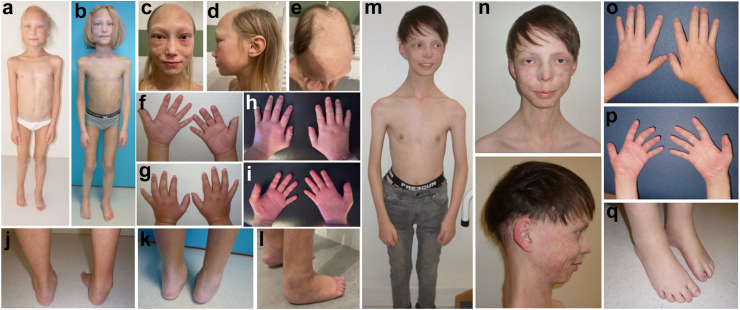
**Clinical course of patients F5 and F1.** Patient F5 carrying the p.(Ser628Asn) variant,^32^ respectively at age 7 (A, F,G, J), age 9 before tenotomy (B, K) and age 12.5 (C, D, E, H, I, L) and patient F1^8^ carrying the p.(Arg627Gly) variant, at age 15 (M, N, O, P, Q), both presenting with poikiloderma, hypotrichosis with patchy alopecia for patient F5 (E), progressive hypotrophy and distal muscle retractions associated with lymphoedema.

Individuals I1, I2, and I3 exhibited the typical clinical features of POIKTMP (Tables 1, S2 and Fig. 1 for the F1^8^ and F5^31^ followed-up patients), namely early-onset poikiloderma (I1, I2, I3), hypotrichosis (I2 and I3) with skin xerosis (I2, I3) and even ichthyosis (I1), exocrine pancreatic insufficiency (I1, I2, I3), lymphoedema of the extremities (I1, I2, I3), as well as muscle damage with contractures (I2, I3) (absent in I1 at the age of 11), palmar hyperkeratosis (I3), heat intolerance (I3), and elevated transaminases (I1).

**Fig. 2:**
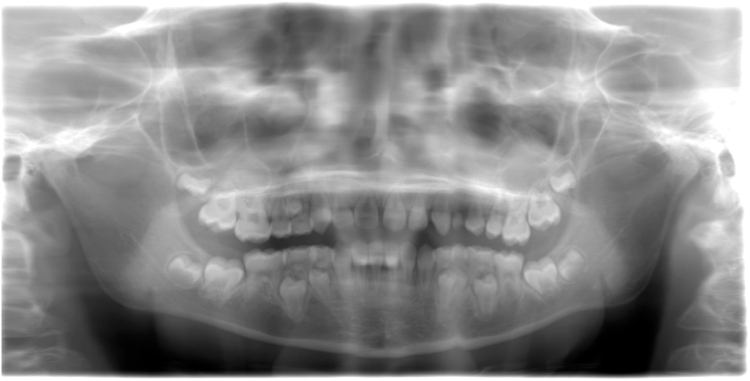
**POIKTMP is associated with dental anomalies.** Orthopantomogram of F5 at age 11 years and 11 months, showing short roots on all teeth, microdontia on 14/24, severe molar incisor hypomineralisation (MIH) on 16, mild MIH on 26/36/46/31/32/21, reduced overlap and overhang, inverted dental articulation on 32/22 and diastema on 11/21.

Clinical signs never or rarely reported before were observed in these 2 families as well as in 5 cases undergoing long-term follow-up conducted by our team: (i) renal failure in F5, also reported in a total of 3 Individuals in the literature review (Table S2), (ii) dental anomalies in F5 such as short, tapered roots on permanent teeth (Fig. 2), also described in another individual,^32^ (iii) hypoparathyroidism in F5, also reported in the previous individual,^32^ (iv) an axonal motor neuropathy detected by electroneuromyogram in I2, aged 47, and a myogenic pattern associated with her myopathy; (v) cholelithiasis at the age of 18 months in I1, for whom a liver biopsy was performed, (vi) splenomegaly in I1, reported in another Individual at the age of 8 months.^30^

In total, based on these 4 new individuals, 5 individuals with long-term follow-up and 32 cases already reported in the literature, the clinical signs of POIKTMP had a frequency of 100% for poikiloderma, 89% for hypotrichosis and hypohidrosis, 74% for myopathy with contractures present in 77% of cases, 64% for moderately elevated serum creatine kinases, and 13% for a severe form of myopathy, 30% for spinal deformity such as scoliosis or rigid spine, 50% for liver damage (mainly elevated transaminases, but acute hepatitis in 8% of cases), 67% for exocrine pancreatic insufficiency and adenocarcinoma in 3 adults (i.e.,; 13% of adults). Failure to thrive was noted in 45% of cases with severe hypotrophy (BMI < 15 in 27% [n = 6/22]). Renal failure, dental and nail anomalies, endocrinological (hypothyroidism and hypoparathyroidism), ophthalmological, haematological disorders were not systematically investigated, and their frequency remains difficult to determine. Importantly, as shown in Supplementary Table S3, no significant differences were observed between males and females regarding age, inheritance patterns, or the prevalence of characteristic clinical features of the disease.

### All *FAM111B* variants cluster in two specific hotspot regions

**Fig. 3:**
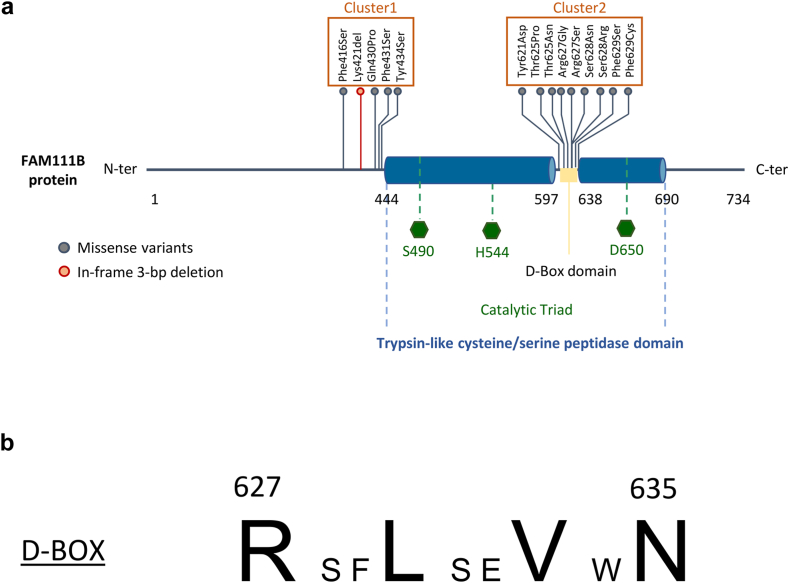
**Distribution pattern analysis of the *FAM111B* variants across the FAM111B full-length protein. a.** Schematic representation of the FAM111B protease primary structure depicting the distribution and localization of the 10 residues subjected to missense mutations and one in-frame deletion identified in POIKTMP so far, as indicated. The catalytic and Destruction (D)-box domain are marked in dark blue and yellow, respectively. The three catalytic triad residues Ser490, His544 and Asp650 are highlighted in green. More than 90% of the pathogenic FAM111B variants are clustered in two regions encompassing amino acids 416–431 and 621–628, as indicated. **b.** D-Box sequence of FAM111B. Residues matching the consensus are shown in bold.

In this study, we describe two previously unreported nucleotide *FAM111B* variants, c.1301A>C and c.1880G>C giving rise to p.(Tyr434Ser) and p.(Arg627Gly) missense protein variants in Families A and B, respectively (Table 1). Interestingly, the p.(Arg627Gly) variant, already associated with POIKTMP, was reported to stem from a distinct nucleotide variant, namely c.1879A>G^8^ (Table 1). In an effort to predict the functional consequences of the genomic alterations of *FAM111B*, we first examined the distribution of the 14 *FAM111B* variants identified so far within the primary structure of the FAM111B protein. As depicted in Fig. 3a, FAM111B features a catalytic region located at the C-terminus, within which resides a destruction (D)-box degron spanning 41 amino acids. Interestingly, around 65% of the *FAM111B* variants were localized within or close to this D-box motif (cluster 2). Notably, one variant impacted Arg627, which serves as a consensus amino acid within the D-box domain (Fig. 3b). Given that D-box sequences are commonly present in substrates of the anaphase-promoting complex (also referred to as cyclosome or APC/C) ubiquitin ligase,^16^ this observation suggests that FAM111B might naturally serve as a substrate or decoy for APC/C that could modulate its activity. This assumption also implies that variants within the D-box region could potentially interfere with FAM111B turnover, and/or APC/C activity and, finally, have an impact on the late mitotic process. The majority of the remaining identified pathogenic variants of FAM111B were clustered in another hotspot located directly upstream of the FAM111B catalytic region (cluster 1), covering residues 416 to 430, which are likely to have an impact on protease activity (Fig. 3a). Taken together, these data indicate that, based on their respective locations, *FAM111B* variants may have distinct effects on FAM111B function, influencing either protein turnover or protease activity.

### Genotype-phenotype correlation: variants located in cluster 2, including the D-box domain are likely associated with a more severe phenotype

We then investigated a potential genotype-phenotype correlation among individuals with a genetic variant located in cluster 1 (group 1) or cluster 2, which included the D-box domain (group 2) (Fig. 3a), as suggested by Arowolo et al.^12^ To this end, we compared the frequency of various clinical signs between the 2 groups. As shown in Supplementary Tables S2 and S4, no significant differences were observed between the two groups, but 5/24 patients presented with severe muscular forms requiring surgery or leading to loss of walking ability (at 3 years in F8)^32^ in group 2 compared to 0/16 patients in group 1 (p = 0.14). Lung impairment was observed in 10 out of 20 patients in group 2, compared to 2 out of 13 patients in group 1 (p = 0.28, Fisher’s exact test). Notably, all three patients diagnosed with pancreatic adenocarcinoma^8^^,^^31^^,^^32^ were in group 2, while no such cases have been reported in group 1 to date (p = 0.26, Fisher’s exact test). Similarly, severe liver damage, including one fatal case at the age of 17, was documented in 3^8^^,^^32^, ^33^, ^34^, ^35^, ^36^, ^37^ out of 24 patients in group 2, whereas no patients in group 1 exhibited this complication (p = 0.28).

### Impact of D-box variants on FAM111B protein expression level

**Fig. 4:**
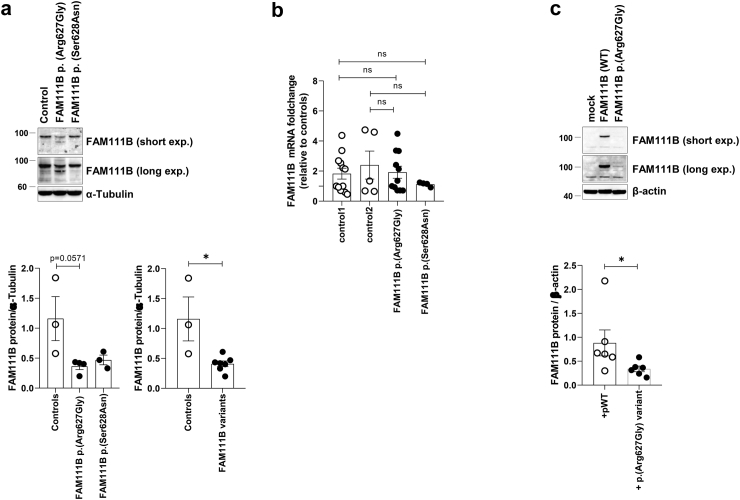
**Impact of the p.(Arg627Gly) and p.(Ser628Asn) variants on FAM111B transcription and protein expression levels. a.** Protein lysates from control and POIKTMP patient fibroblasts were separated by SDS-PAGE prior to Western-blotting using antibodies specific for FAM111B and α-tubulin which served as a loading control, as indicated (shown is one representative experiment and two exposure times). In the lower panel, both FAM111B and α-tubulin immunoreactive bands were quantified by densitometry and the FAM111B values were normalized to those of α-tubulin. Data are presented as mean (±SEM) of FAM111B to α-tubulin protein ratios from at least 4 independent experiments for each group. Statistical significance was assessed by unpaired Student’s t-test (∗p < 0.05). **b.** Total RNA extracted from control and two POIKTMP patient fibroblasts with p.(Arg627Gly) or p.(Ser628Asn) variants were subjected to RT-qPCR using primers for *FAM111B* as well as PPIA and RPL13A (housekeeping genes). Shown are the fold-change values of FAM111B mRNA in the control and patient groups relative to one calibrator control expressing wild-type *FAM111B* whose relative quantifications were set to 1. Columns represent means ± SEM from at least 7 experiments for each group. **c.***FAM111B*^−/−^ BHK-21 cells were transfected with wild-type (pWT) or p.(Arg627Gly) *FAM111B* constructs for 24 h prior to protein extraction and SDS-PAGE/Western-blot analysis using antibodies directed against FAM111B and β-actin (loading control). Two exposure times are shown, as indicated. Densitometry analysis was used for quantification of the immunoreactive bands followed by normalization of the FAM111B intensity values to those of β-actin. Data are presented as mean (±SEM) of FAM111B to β-actin protein ratios from 6 independent experiments for each group. Statistical significance was assessed by Mann–Whitney U test (∗p < 0.05).

Due to the clustering of *FAM111B* variants within or near the D-box degron motif (Fig. 3a), our next objective was to assess how these variants affect the FAM111B steady-state protein expression level. To address this point, we undertook a comparative examination of FAM111B protein contents in cells isolated from healthy individuals and patients with POIKTMP using SDS-PAGE/Western-blotting. Our data revealed that POIKTMP patient cells carrying the p.(Arg627Gly) or the previously reported p.(Ser628Asn)^8^ *FAM111B* variants exhibited reduced levels of FAM111B protein expression compared to those of wild-type cells (p = 0.012, Student’s t-test) (Fig. 4a). Crucially, the levels of *FAM111B* transcripts exhibited minimal variation between control and POIKTMP patient samples (Fig. 4b), indicating that the diminished protein pools resulting from the p.(Arg627Gly) or p.(Ser628Asn) substitutions were attributed to increased protein degradation but not reduced gene expression. This notion was further substantiated by introducing the p.(Arg627Gly) *FAM111B* mutant into BHK-21 cells that do not express endogenous FAM111B. Indeed, although supplying mRNA levels comparable to wild-type *FAM111B*, the p.(Arg627Gly) *FAM111B* mutant led to a significant reduction (p = 0.026, Mann–Whitney U test) in FAM111B protein expression (Fig. 4c).

### The p.(Arg627Gly) FAM111B protein variant does not exhibit a shorter half-life than its wild-type counterpart but follows a distinct degradation pathway

**Fig. 5:**
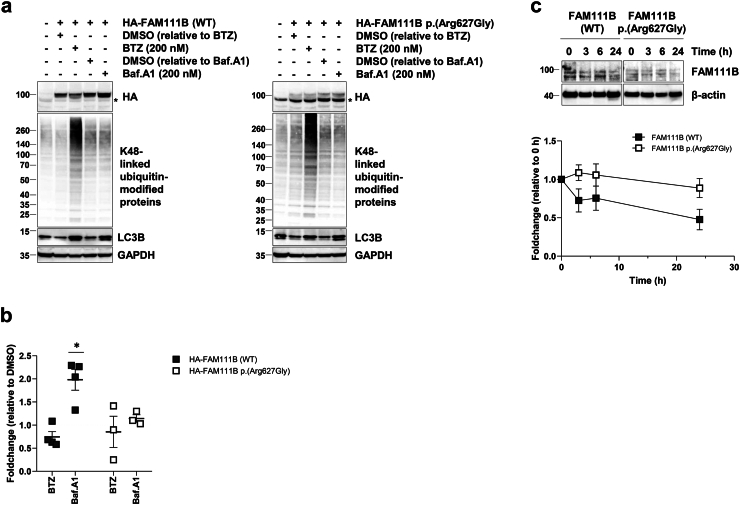
**Effects of proteasome and autophagy inhibition on the steady-state expression of wild-type and p.(Arg627Gly) FAM111B. a.** HeLa cells transiently expressing N-terminally HA-tagged wild-type (WT) or p.(Arg627Gly) FAM111B were treated with bortezomib (BTZ) or bafilomycin A1 (Baf.A1) for 12 h prior to protein extraction and SDS-PAGE/Western blot analysis. Membranes were probed with antibodies against HA, K48-linked ubiquitin chains, LC3B, and GAPDH (loading control), as indicated. Vehicle controls consisted of DMSO at equivalent volumes used for BTZ and Baf.A1 treatments. An asterisk indicates a nonspecific band. Shown is one representative experiment out of three. **b.** Densitometric quantification of HA-immunoreactive bands from Western blots in (A). Data are presented as fold-change mean values ± SEM relative to DMSO controls from at least three independent experiments. Statistical significance was determined using a paired t-test (∗p < 0.05). **c.** SDS-PAGE/Western blot analysis of control (FAM111B WT) and patient-derived (FAM111B p.Arg627Gly) fibroblasts treated with cycloheximide (50 μg/mL) for the indicated time points. Blots were probed with antibodies against FAM111B and β-actin (loading control). Lower panel: Densitometric quantification of FAM111B bands from three independent experiments. Data are presented as fold-change values (mean ± SEM) relative to untreated cells (0 h).

To explore the mechanisms underlying FAM111B protein degradation, HeLa cells were transfected with an HA-tagged version of either wild-type or p.(Arg627Gly) FAM111B. After transfection, cells were treated for 12 h with bortezomib (BTZ) to inhibit proteasome-mediated breakdown or bafilomycin A1 (Baf.A1) to block autophagy-mediated degradation. As shown in Fig. 5a and b, BTZ induced the accumulation of ubiquitinated proteins but did not stabilize either wild-type or mutant FAM111B, suggesting that neither variant undergoes degradation via the UPS. In contrast, autophagy inhibition with Baf.A1, while disrupting autophagic flux as evidenced by the absence of LC3B stabilization, led to a significant increase in the steady-state levels of wild-type FAM111B (p = 0.025, Student’s t-test) (Fig. 5a and b). Intriguingly, this effect was not observed for the p.(Arg627Gly) variant. These findings suggest that the Arg627Gly substitution impaired the targeting of FAM111B to autophagosomes. Surprisingly, although the steady-state levels of mutant FAM111B were lower than those of its wild-type counterpart, its half-life was not reduced (Fig. 5c). This observation suggests that the mutant protein was not particularly prone to degradation and that its reduced levels may result from translation inefficiency. Collectively, these findings indicate that variants affecting the D-box degron of FAM111B impaired both its translation efficiency and degradation pathway.

### *FAM111B* D-box missense variants are associated with transcriptional changes in fundamental cellular processes in POIKTMP patient cells

**Fig. 6:**
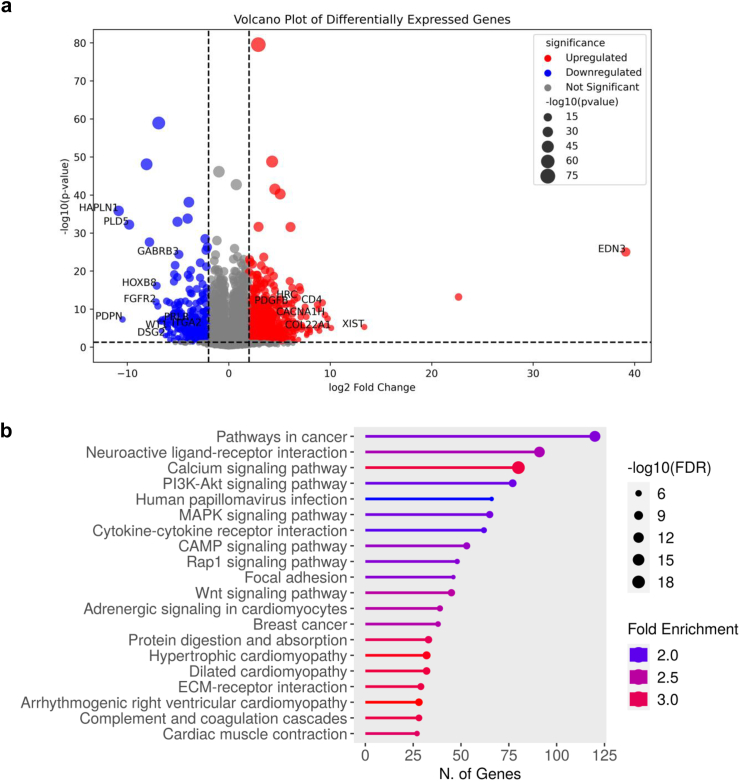
**Transcriptomic analysis of fibroblasts from healthy individuals versus POIKTMP patient-derived fibroblasts carrying p.(Arg627Gly) or p.(Ser628Asn) FAM111B variants. a.** Total RNA was extracted from two fibroblast cell lines derived from healthy donors and two fibroblast cell lines from POIKTMP patients carrying either the p.(Arg627Gly) or p.(Ser628Asn) FAM111B variant. RNA-seq was performed, and differentially expressed genes (DEGs) were visualized using a volcano plot. The x-axis shows the log2 fold change in gene expression, with positive values indicating upregulation and negative values indicating downregulation. The y-axis represents the −log10 (p-value), reflecting the statistical significance of each gene's differential expression. Red dots indicate upregulated genes, blue dots denote downregulated genes, and grey dots represent genes that are not significantly differentially expressed. Dot size correlates with the significance level, with larger dots representing higher significance based on their p-values. **b.** KEGG pathway enrichment analysis of differentially expressed genes (DEGs) was performed using ShinyGO 0.8. Pathways are ranked by the number of deregulated genes involved in each pathway. The x-axis represents the number of genes, while the colour gradient reflects fold enrichment, with red indicating the highest enrichment. Dot size corresponds to the -log10 of the false discovery rate (FDR), with larger dots indicating more statistically significant pathways.

To investigate the transcriptomic consequences of *FAM111B* missense variants, we performed RNA-seq analysis on fibroblasts derived from POIKTMP patients carrying the Arg627Gly or Ser628Asn heterozygous substitutions and compared them to control fibroblasts expressing wild-type *FAM111B*. Volcano plot analysis of differentially expressed genes (DEGs) identified significant transcriptional changes, with many of the most dysregulated genes involved in extracellular matrix (ECM)-receptor interaction (e.g., *HAPLN1*, *COL22A1*, *DSG1*, *ITGA2* and *PDPN*) and cell signalling (e.g., *PLD5*, *FGFR2*, and *PDGFB*) (Fig. 6a). Overlaying the transcriptional changes onto functional groups using KEGG database^38^, ^39^, ^40^ revealed clustering of DEGs into 20 pathways (Fig. 6b). Our analysis confirmed that among these pathways, those potentially relevant to POIKTMP pathogenesis included calcium signalling, PI3K-Akt signalling, and cytoskeletal dynamics, with notable alterations in extracellular matrix-receptor interactions and focal adhesions (Fig. 6b).

### Proteomic changes associated with *FAM111B* D-box missense variants unveil UPS remodelling

To strengthen our analysis regarding the impact of *FAM111B* missense variants on cellular activity, we performed quantitative proteomic analysis using nanoscale liquid chromatography coupled to tandem mass spectrometry (Nano LC-MS/MS). Our data revealed that *FAM111B* variants were associated with changes of the expression levels of numerous proteins. Prime examples of these alterations included the upregulation of integrin ITGA3 as well as interferon-inducible gene (ISG) products such as STING and IFITM3, alongside the downregulation of the EPB41L3 protein, consistently observed across three independent experiments (Supplementary Fig. S1). Similar to the transcriptomic analysis, we then sought to gain deeper insights into the proteomic consequences of *FAM111B* variants by classifying differentially abundant proteins (DAPs) into functional groups using KEGG pathway analysis. Strikingly, and in line with the transcriptomic findings, focal adhesion, ECM-receptor interaction, and PI3K-Akt signalling pathways were consistently affected across all three independent experiments (Supplementary Fig. S1).

**Fig. 7:**
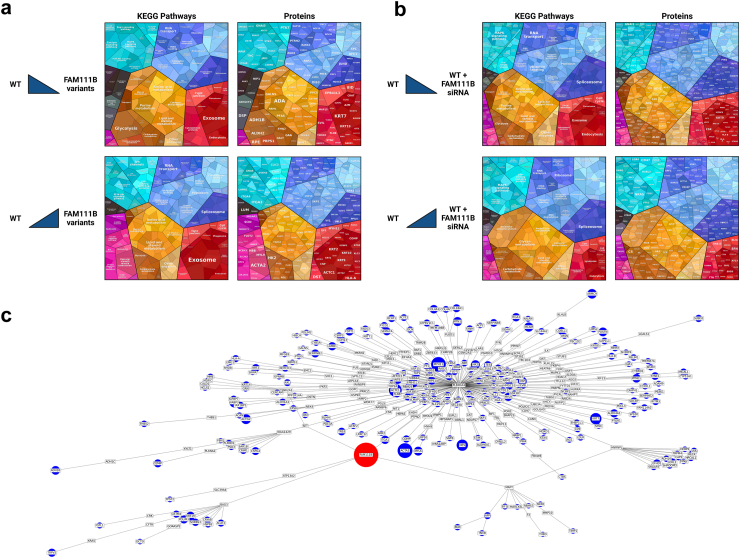
**Proteomic comparative analysis of control cells, POIKTMP patient cells with p.(Arg627Gly) or p.(Ser628Asn) variants, and siRNA mediated FAM111B-depleted cells. a.** Protein lysates from samples described in Fig. 6 were analysed Nano LC-MS/MS and the differentially abundant proteins (DAPs) were depicted by proteomaps. The upper and lower panels illustrate the involved KEGG pathways and proteins that display reduced and elevated abundance (t-test p-value ≤ 0.05; min fold-change of 2), respectively, in FAM111B mutant compared to wild-type fibroblasts. Functionally related pathways and proteins are arranged in common regions and coded using similar colours. **b.** Shown are the proteomaps showcasing the DAPs with decreased abundance (upper panel) or increased abundance (lower panel) in fibroblasts exposed to FAM111B siRNA compared to fibroblasts treated with scrambled siRNA (t-test p-value ≤ 0.05; min fold-change of 2), as indicated. **c.** Interaction network delineating the connections between FAM111B and the identified DAPs in mutant versus wild-type FAM111B fibroblasts. The red node symbolizes the target protein (FAM111B), while the blue nodes represent differentially abundant proteins (DAPs), their size corresponding to relative abundance (fold change). Intermediate proteins are denoted by white blocks in the representation. The interaction graph was generated using a comprehensive tool that integrates node embedding, deep walk techniques, and the nearest neighbour algorithm.

To further explore and identify subtle proteomic alterations, we generated proteomaps to cluster DAPs based on their KEGG pathway annotations. This analysis revealed that, in addition to pathways related to cell signalling and cytoskeletal dynamics, numerous DAPs were linked with processes such as metabolism, RNA transport, endocytosis and protein degradation by the ubiquitin-proteasome system (UPS) (Fig. 7a). Importantly, the proteomic changes observed in cells carrying *FAM111B* variants closely mirrored those observed in *FAM111B*-silenced cells (Fig. 7b), although the specific DAPs and their corresponding KEGG pathways did not always overlap (Supplementary Fig. S2). Indeed, remodelling of the UPS was more pronounced in *FAM111B*-depleted cells, with several of the most significantly regulated DAPs associated with this pathway (Supplementary Fig. S2b and c).

To further integrate these findings, we performed a consolidated analysis of transcriptomic and proteomic datasets to identify proteins commonly dysregulated in both *FAM111B*-mutant and *FAM111B*-silenced cells. As shown in Table 2, this analysis confirmed that many of the overlapping proteins were associated not only with cell signalling (e.g., AKT1, EPB41L3, ARGHGEF2) or cytoskeletal dynamics (ITGA3, FLNA, LIMA1) but also with the UPS, as highlighted by a keyword-based analysis of statistically significant DAPs. Notably, these included proteasome α-subunits such as PSMA2 and PSMA5, as well as key components of the ubiquitin-like modifier ISG15 conjugation machinery (e.g., ISG15, UBA7, and TRIM25) (Table 2). Interestingly, the upregulation of the ISG15 modification pathway, typically induced by interferons (IFN),^41^ along with the enrichment of other IFN-inducible gene products, including IFIT1, MX1, and IFITM3 (Table 2), strongly supported the presence of a type I IFN signature associated with *FAM111B* dysfunction. Collectively, these findings suggest that *FAM111B* alterations drive profound changes in cell signalling, cytoskeletal dynamics, the UPS, and innate immune responses, all of which may contribute to the molecular pathogenesis of POIKTMP.

**Table 2:** Common DAPs (fold change > 0.5, p-value < 0.05) identified in differential proteomic analyses of *FAM111B*-mutated and *FAM111B*-silenced fibroblasts compared to wild-type FAM111B.

Keywords	Common DAPs	Biological Processes	KEGG pathways
26S proteasome	**PSMA2; PSMA5; PSMB6; PSMD3; PSMD4; PSME1; PSME3**	Cell differentiation; Localization; Reg. of transport; Reg.of stimulus; Reg. of Molecular function	Focal adhesion; ECM-receptor interaction; PI3K-AKT signalling; Apoptosis
Ubiquitin	**TRIM22; ISG15; UCHL1; XIAP; USP15; UBE2D1; UBE2D3; RNF213; TRIM25; TRIM21; UBE2M; ITCH; UBA7**		
Integrin protein	**ITGA3; ITGA4; ITGA1; ITGA7; ITGB3**		
Filamin	**FLNB; FLNC; FLNA; FBLIM1; FILIP1L**	Cell differentiation; Localization; Reg. transport	Focal adhesion; ECM-receptor interaction; Myofibrill
Cytokine Junction	**LAMB1; VIM; FBN1**		
GAP junction	**GJA1**		
Junction, Migration	**SYNPO2; CEMIP; LRP1; COMP; TUBB4A; SVIL**		
Cytoplasmic Dynein	**DYNC1H1; DYNC1LI1; FSTL1**		
SWI/SNF related Matrix associated actin	**SMARCD2; SMARCAS**		
MYOSIN family	**MYL9; MYLK; MYH11; MYH10; MYO1B; TMP2**		
LIM Domain protein	**LIMA1; LMCD1; FHL1; LIMCH1; LIMD1**		
Serine/Threonine Kinase	**AKT1; DCLK1; STK4; STK3; PPP3CC; PAK1; PPP2CA; PPP2CB; CDC42BPA; NEK7; PKN1; PLPP3**	Cell differentiation; Localization; Reg. of stimulus	PI3K-AKT signalling; Signalling Pathways
EH domain containing	**EHBP1; EHBP1L1**	Cell differentiation; Localization; Reg. of stimulus	ECM-receptor interaction; Signalling Pathways; Cell Cycle
Band 4.1 like protein	**EPB41L3; EPB41L2**		
Signal transducer	**STAT1; STAT2; LRRC40**		
RHO protein Family	**RHOB; MPRIP; ARGHGEF2; ROCK2; SRGAP2; NRBP1**		
POTE ankyrin domain	**POTEI; POTEP, POTEJ**		
FERM domain protein	**FRMD6; FRMD8; FERMT2**		
Interferon	**IFITM3; IFIT3; IFIT5; MX1; MX2; IFIH1; IFIT1; IFIT2; STING1; DPP7**	Reg. of stimulus; Immune system	Signalling Pathways
Growth factor	**IGFBP3; IGF2BP3, IGF2BP1; IGFBP7; EGFR; EPS8; IGF2R**	Cell differentiation; Immune system	Signalling Pathways
DNA/RNA binding	**H2AC21; H2AC6; H2AC8; RBSM1; RBSM2;RBSM3; DDX3Y; DDX1; DDX6**	Cell differentiation	RNA in cancer

DAPs were categorized using relevant keywords and mapped to their associated KEGG pathways and biological processes (FDR < 0.05, sorted by fold enrichment).

### In silico mapping of connections between FAM111B and identified DAPs

We next employed a bioinformatics tool leveraging node embedding techniques to establish connections between FAM111B and the DAPs. Using STRING's extensive protein-protein interaction network, we created a high-dimensional graph representation of the interactome and then applied node embedding algorithms to uncover latent protein representations within this network. As shown in Fig. 7c, we pinpointed a cluster of connectors linking most DAPs to FAM111B. This group notably included PLEKHA4, BAG3, and ATP13A2, all of which have been linked to ubiquitination processes.^42^, ^43^, ^44^ By integrating vector similarity and nearest-neighbour algorithms, we identified intermediary proteins bridging FAM111B to DAPs, shedding light on novel protein interaction pathways. Accordingly, our analysis revealed the proximity of FAM111B to intermediate proteins, primarily consisting of enzymes associated with the ubiquitin and/or ubiquitin-like conjugation system (Fig. 7c). Our node embedding algorithm has revealed that the intermediary proteins linking FAM111B to DAPs and serving as crucial nodes connecting more than three proteins are predominantly associated with the biological process of “Protein Ubiquitination” (False Discovery Rate, FDR = 0.0102), as determined through analysis in the STRING database. Furthermore, these intermediary proteins exhibit molecular functions such as “Chaperone Binding” and “Ubiquitin Protein Transferase Activity” (FDR = 0.0462), in addition to being associated with annotated keywords related to the “UBL Conjugation Pathway” (FDR = 0.00075). This list of proteins, which we referred to as intermediary nodes, connected more than three proteins (Supplementary Fig. S3a) and was primarily composed of E3 ligases or proteins closely linked to the UPS or quality control protein processes. Moreover, the discovery of APC/C as a pivotal protein, acting as a bridge among virtually all DAPs (Supplementary Fig. S3b), strongly supports the hypothesis that FAM111B serves as a key regulator of the UPS.

### FAM111B indirectly engages in physical interactions with components of the UPS

**Fig. 8:**
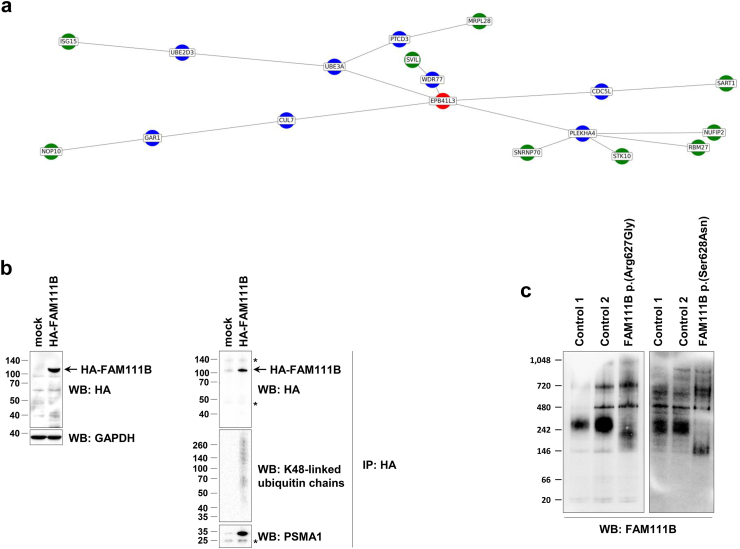
**Quantitative analysis of the FAM111B interactome through SPR-MS, immunoprecipitation and native-PAGE. a.** Proteins captured by FAM111B antibody from whole-cell lysates of both control fibroblasts and fibroblasts derived from a POIKTMP patient with the p.(Arg627Gly) FAM111B variant were enzymatically digested using trypsin prior to the subsequent MS analysis of the resulting peptides. Shown is an interaction network depicting the close proximity between potential interactors of FAM111B variants identified by SPR-MS. Red nodes represent the central protein with a minimum score calculated from the nearest neighbour algorithm, blue nodes represent the intermediate protein, and green nodes represent DAPs or potential interactors. **b.** Whole cell lysates (WCL) from HeLa cells transfected with empty vector (mock) or a plasmid encoding a HA-FAM111B fusion protein were assessed for their content by SDS-PAGE/Western-blotting using antibodies specific for HA and GAPDH (loading control), as indicated. Right panel, HeLa samples were subjected to immunoprecipitation using anti-HA magnetic beads with eluted fractions being analysed by SDS-PAGE/Western-blotting with antibodies direct to HA, K48-linked ubiquitin chains and PSMA1, as indicated. **c.** Whole-cell protein lysates from control fibroblasts and POIKTMP patients carrying the p.(Arg627Gly) or p.(Ser628Asn) FAM111B variant were separated by 3–12% native-PAGE and subsequently analysed by Western-blotting using an antibody specific for FAM111B, as indicated.

We next aimed to decipher the causal relationship between FAM111B loss of expression and the UPS proteomic changes by attempting to identify FAM111B interaction partners. To this end, we employed surface plasmon resonance coupled with mass spectrometry (SPR-MS). Specifically, we conducted ligand fishing experiments on fibroblasts lysates derived from healthy individuals and POIKTMP patients carrying *FAM111B* missense variants, using an antibody directed against FAM111B as a capturing protein. Our subsequent analysis of the isolated protein-ligand complexes by MS identified several putative interaction partners of FAM111B including EPB41L3, SVIL, ISG15, MRPL28, STK10, NOP10, SART1, RBM27, and NUFIP2 (Fig. 8a).

Since, apart from ISG15, none of these proteins were direct core members of the UPS, we further explored the relevance of these candidates using bioinformatic tools similar to ‘node embeddings’ to uncover central and intermediate proteins that could explain the proximity and relationships between potential interaction partners of FAM111B. Interestingly, our data revealed a predominant presence of components of the UPS, notably ubiquitin-protein ligase E3A (UBE3A), E3 ubiquitin-protein ligase E3D (UBE2D3), Cullin-7 (CUL7), Cell Division Cycle 5 Like (CDC5L) among the intermediate proteins, depicted as blue nodes in Fig. 8a. Remarkably, EPB41L3 (red node), stood out as the central protein potentially elucidating the relationships between all statistically identified proteins.

To validate these in silico predictions and determine whether FAM111B interacts with the UPS in a cellular context, a HA-tagged version of wild-type *FAM111B* was introduced into HeLa cells, followed by HA-immunoprecipitation and SDS-PAGE/Western blot analysis. Our results demonstrate that wild-type HA-FAM111B co-precipitated with both K48-linked ubiquitin-modified proteins and the proteasome subunit PSMA1 (Fig. 8b), suggesting physical interactions between these components. Given the molecular weight of the 20S proteasome core particle containing PSMA1 (approximately 700 kDa), these findings indicate that FAM111B was capable of associating with a large protein complex. To further confirm the existence of FAM111B within a protein complex, we next analysed its migration profile under non-denaturing conditions using native-PAGE/Western-blotting. Our data revealed that FAM111B from protein lysates extracted from control cells exhibited a strong migration pattern at approximately 300 kDa (Fig. 8c), higher than the predicted 84.5 kDa molecular weight, thereby confirming that FAM111B integrates a high molecular weight protein complex. Most interestingly, the migration profile of protein lysates extracted from POIKTMP patient cells with p.(Arg627Gly) or p.(Ser628Asn) *FAM111B* variants shifted downward (Fig. 8c), suggesting the loss of one or more interacting partners. Taken together, these data suggest that FAM111B interacted with UPS components and that such interactions were lost in POIKTMP patient cells.

### *FAM111B* D-box missense variants perturb UPS function and consequently lead to the disruption of protein homoeostasis

**Fig. 9:**
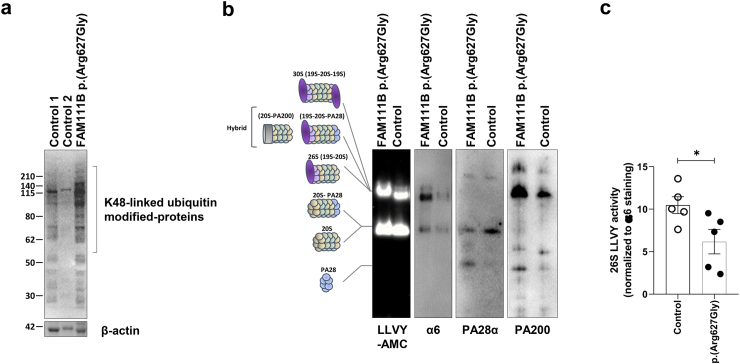
**Assessment of the ubiquitin expression profile and proteasome function in POIKMP patient cells carrying the p.(Arg627Gly) *FAM111B* pathogenic variant. a.** RIPA cell lysates from fibroblasts derived from healthy donors (controls 1 and 2) or POIKTMP patient with p.(Arg627Gly) FAM111B variant were separated by SDS-PAGE prior to Western-blot analysis using antibodies directed against K48-linked ubiquitin chains and β-actin (loading control), as indicated. **b.** Non-denatured protein lysates from control and POIKTMP patient fibroblasts carrying a p.(Arg627Gly) FAM111B variant were separated by 3–12% native-PAGE and proteasome bands were visualized by in-gel overlay assay using 0.1 mM of the fluorogenic Suc-LLVY-AMC peptide, as indicated. Gels were further blotted onto membranes for Western-blot analysis using antibodies specific for α6, PA28α, and PA200, as indicated. The schematic to the left depicts the proteasome complexes (30S, hybrid, 26S, 20S-PA28 and 20S) and free regulators (PA200 and PA28) detected by the three antibodies. **c.** Fluorescent (AMC) 26S proteasome bands were quantified by densitometry and normalized to those stained with the α6 antibody. Shown are the fold change values of AMC intensity (proteasome activity) of the 26S proteasome complexes in POIKTMP patient cells harbouring the p.(Arg627Gly) FAM111B variant versus control cells whose densitometric measurements were set to 1. Statistical significance was assessed by Mann–Whitney U test (∗p < 0.05).

One major role of the UPS is to preserve protein homoeostasis by tagging damaged and/or non-functional proteins with ubiquitin molecules before targeting them to proteasome-mediated degradation.^45^ The finding that in POIKTMP patient cells, the association of FAM111B with complexes involving UPS components is weakened (Fig. 8c), suggested a potential alteration of protein homoeostasis in these cells. To answer this question, we monitored the levels of ubiquitin-modified proteins in POIKTMP patient cells using SDS-PAGE/Western-blotting. As shown in Fig. 9a, the p.(Arg627Gly) *FAM111B* variant was associated with increased accumulation of ubiquitin-protein conjugates, suggesting that *FAM111B* variants perturbed protein homoeostasis, as a consequence of either increased ubiquitin conjugation or decreased breakdown of ubiquitinated proteins.

To address whether the observed disruption of protein homoeostasis in POIKTMP patient cells was caused by proteasome dysfunction, we next assessed the chymotrypsin-like activity originating from proteasomes in these samples using an in-gel overlay assay, as previously described.^46^ As shown in Fig. 9b and Supplementary Fig. S4, the activity of the various proteasome complexes did not substantially change between control and patient cells. However, subsequent staining of the gel with antibodies specific for α6, PA28α and PA200 revealed significant disparities in the composition of proteasome complexes between control and POIKTMP patient cells. Indeed, cells with p.(Arg627Gly) *FAM111B* variant exhibited increased amounts of 30S, 26S and hybrid proteasomes as compared to control cells (Fig. 9b). This correlated with an augmented interaction of 26S complexes with PA200 and a reduced association of 20S complexes with PA28α/β (Fig. 9b). Importantly, because control and POIKTMP cells expressed comparable levels of α6, PA28α and Pb200 individual proteins (Supplementary Fig. S5), the changes in proteasome complexes associated with *FAM111B* variants were likely to reflect variations in proteasome assembly and/or binding capacities to the regulators PA200 and PA28α/β. Strikingly, normalizing proteasome activity to the amounts of 30S, 26S and hybrid complexes revealed that chymotrypsin-like activity was lower in POIKTMP patient cells than that of control cells (p = 0.0317, Mann–Whitney U test) (Fig. 9c). These data indicate that POIKTMP proteasomes were less efficient than their wild-type counterparts. Altogether, these findings demonstrate that both ubiquitin-conjugation system and 26S proteasomes were dysregulated in POIKTMP patient cells, which ultimately led to an imbalance in protein homoeostasis.

### POIKTMP patient cells with *FAM111B* D-box missense variants express typical biomarkers of protein homoeostasis perturbations

To further ascertain the notion that *FAM111B* missense variants compromise UPS function, we next search for expression of biomarkers known to be induced under these conditions. Key indicators of impaired UPS activity include increased autophagy and the development of type I IFN gene signatures.^47^^,^^48^ To address this aspect, we assessed autophagy flux in both control and POIKTMP patient cells by monitoring the steady-state expression level of the autophagosome-associated protein LC3B-II. For this purpose, we measured LC3b-II levels in the presence of the vacuolar proton pump inhibitor Baf.A1 to clarify the possible interpretations of increased or decreased levels of LC3-II.^49^ As shown in Supplementary Fig. S6, although some variability was observed across experiments, no discernible differences were detected in the levels of LC3B-II between Baf.A1-treated control and patient-derived fibroblasts carrying the p.(Arg627Gly) *FAM111B* variant, suggesting this substitution did not substantially alter the autophagic flux.

**Fig. 10:**
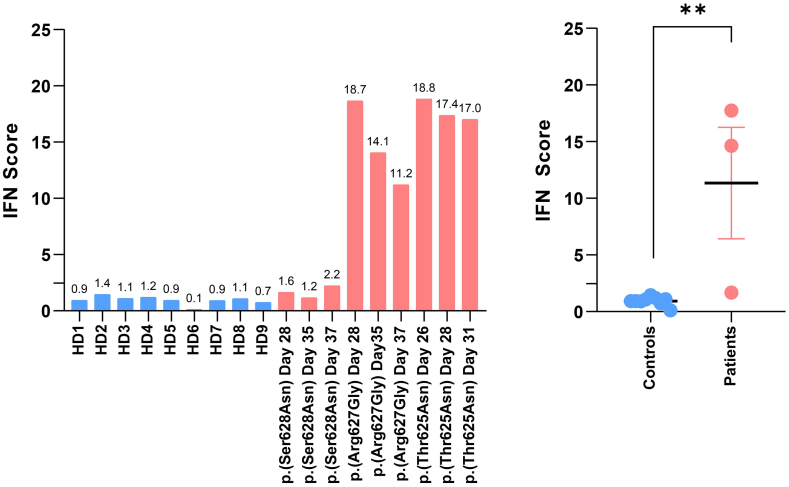
**Analysis of autophagy and IFN-related biomarkers in POIKTMP patient cells with p.(Arg627Gly), p.(Ser628Asn) or p.(Thr625Asn) FAM111B variant.** Total RNA was extracted from T cells derived from nine healthy donors (HD) and three POIKTMP patients with p.(Arg627Gly), p.(Ser628Asn) or p.(Thr625Asn) FAM111B variants after various days of culture were subjected to RT-qPCR for gene expression analysis of IFN-stimulated genes (IFIT1, IFI27, IFI44, IFI44L, ISG15, and MX1), as indicated. Shown are the individual (left) and grouped (right) IFN scores which were calculated as the median of the relative quantifications of the six ISG over a single HD which was used as calibrator control for each sample. Statistical significance was assessed by Mann–Whitney U test (∗∗p < 0.01).

Controls and POIKTMP patient samples [p.(Arg627Gly), p.(Ser628Arg) and p.(Thr625Asn)] were also subjected to RNA extraction and subsequent quantitative RT-PCR to measure the transcription of type I IFN-stimulated genes (ISG) including *IFIT1*, *IFI44*, *IFI44L*, *IFI27*, *ISG15*, and *MX1*. The transcription rate of the six examined ISG was elevated in T cells carrying a p.(Arg627Gly) and p.(Thr625Asn) *FAM111B* variant compared to control T cells expressing wild-type *FAM111B* (Figs.10 and S7). Calculation of type I IFN scores employing the ISG fold change over unrelated healthy controls and following the procedure of Rice et al.^50^ revealed that two out of the three *FAM111B* variants were associated with substantial type I IFN responses (p = 0.0091, Mann–Whitney U test) (Fig. 10). Altogether, these findings reinforce the notion that cells from POIKTMP patients with *FAM111B* D-box missense variants engaged a program designed to compensate for UPS dysfunction and restore protein homoeostasis by upregulating autophagy and triggering type I IFN responses.

## Discussion

In this study, we confirmed the multi-systemic nature of POIKTMP and specified the frequency of the main clinical signs in a cohort of 41 individuals, including 4 new individuals from 2 independent families as well as 37 previously reported cases (Table 1). Previously unreported clinical signs were observed in several individuals and were most likely part of the clinical spectrum of POIKTMP such as (i) renal failure, described in 3 individuals (including 2 children), (ii) dental anomalies, in particular dental agenesis and root anomalies and (iii) hypoparathyroidism, both described in 2 individuals. Regarding neuropathy diagnosed in one adult individual (I3), it is likely that it occurs mainly in adults in POIKTMP and as our cohort is predominantly paediatric (n = 18), it is conceivable that this complication is part of POIKTMP but has not yet been observed. In addition, electroneuromyogram (ENMG) was not performed in all individuals. Ultimately, to determine whether the occurrence of cholelithiasis (I1) and splenomegaly (I1; F2 (22)) in early childhood is indeed part of the clinical spectrum of POIKTMP, additional data will be required. Hence, in addition to the standard clinical monitoring typically recommended for these individuals,^10^ we advocate vigilance in monitoring renal and parathyroid functions, regular dental assessments, and consideration of EMG if clinical uncertainty arises regarding neuropathy. Overall, the multisystemic involvement of POIKTMP underscores the necessity for meticulous and comprehensive monitoring. Besides, while this cohort represents the largest collection of POIKTMP cases reported to date, the limited number of cases may still pose challenges in determining whether the cohort fully captures the complete clinical and genetic spectrum of POIKTMP. Indeed, the sample size in our study is inherently limited due to the ultra-rare nature of POIKTMP. One should therefore emphasize that the full extent of the clinical spectrum remains incompletely understood, with certain clinical features reported in only a single patient, raising the possibility of coincidental associations. An essential component for gaining a better understanding of the natural history of the disease and adapting the management of affected individuals is a long-term follow-up.

At the genetic level, we reported in this study two new nucleotide variants out of the 11 different variants identified so far, giving rise to a total of 13 different protein variants. These variants were in two hot-spot regions, namely in the D-box domain and in the FAM111B protease catalytic region, potentially exerting distinct functional effects. A comprehensive analysis of individuals with a variant in cluster 1 of *FAM111B* versus cluster 2 suggested that cluster 2 variants were associated with a more severe phenotype, characterized by muscle, lung, and liver damage, as well as a predisposition to pancreatic cancer (Supplementary Table S2). However, it is essential to note that this observation could not be statistically confirmed, although it is consistent with a previous study.^12^ Again, a study involving a larger cohort of individuals is necessary to further advance research on this ultra-rare disease.

Our functional analysis highlighted that *FAM111B* variants perturbed the UPS (Fig. 9), while exerting notable effects on cell signalling, cytoskeletal dynamics, and immune responses (Fig. 7 and Supplementary Fig. S1). These findings align with the clinical manifestations of POIKTMP, including impaired tissue integrity, inflammation, and dysregulated cellular proliferation. The strong overlap between the transcriptomic and proteomic data (Table 2) underscored the central role of FAM111B in maintaining cellular homoeostasis through regulation of the UPS and associated pathways.

At the cellular level, we showed that variants affecting the FAM111B D-box domain resulted in reduced FAM111B protein contents (Fig. 4, Fig. 5a), which was associated with a remodelling of essential UPS components (Fig. 7, Fig. 9). The decreased FAM111B protein levels, as observed in the presence of the Arg627Gly substitution initially suggested that these variants might disrupt the native conformation of the full-length protein, leading to the production of defective ribosomal products (DRiPs), as previously described.^51^^,^^52^ However, proteasome inhibition failed to rescue p.(Arg627Gly) FAM111B, which would be expected if this variant was indeed associated with the production of DRiPs. These results suggested that the Arg627Gly substitution did not destabilize FAM111B, and the reduced protein levels of this variant in cells may be attributed to other factors. Notably, the p.(Arg627Gly) variant was not degraded more rapidly than its wild-type counterpart (Fig. 5c), indicating that it was not particularly prone to protein breakdown. Instead, the low protein content associated with this variant was likely due to decreased protein synthesis, possibly resulting from a deleterious effect of the Arg627Gly substitution on FAM111B translation efficiency.

Our analysis aimed at connecting the FAM111B protein to the identified DAPs suggests that APC/C may act as a critical hub, potentially linking multiple DAPs (Supplementary Fig. S3). A potential role for APC/C in POIKTMP pathogenesis was further strengthened by the observation that APC/C variants lead to the development of RTS type 1, a disease characterized by symptoms remarkably similar to those of POIKTMP.^3^^,^^4^ Considering the role of APC/C as a ubiquitin ligase regulating cell cycle,^53^ these findings pointed to cell proliferation as a key factor driving POIKTMP pathogenesis. Besides, the APC/C complex is renowned for its ability to target cell cycle-related proteins for degradation, thereby regulating the eukaryotic cell cycle, particularly during anaphase entry and mitotic exit.^54^, ^55^, ^56^ In this regard, a recent study demonstrates that the FAM111B protein localizes within the centrosome and mitotic spindles,^57^ suggesting that FAM111B might exert its effect on cell proliferation through APC/C. However, exploring the underlying molecular mechanisms could prove challenging, given that APC/C interactions extend beyond protein breakdown to also include protein stabilization.^58^ These many roles add complexity to our understanding of how APC/C influences POIKTMP.

To date, there is limited knowledge about the physiological role of FAM111B and the mechanisms behind POIKTMP molecular pathogenesis. Previous data, primarily derived from studies on the homologous protein FAM111A, strongly suggested the involvement of FAM111B in cell cycle regulation. In this work, we not only corroborated this notion but also suggested additional crucial cellular functions. However, a key limitation of this study was the small number of POIKTMP patient samples (n = 2, three independent replicates) available for omics analyses, a challenge inherent to the ultra-rarity of the disease and the limited availability of biological material. To address this, we performed parallel experiments in healthy cells using siRNA-mediated downregulation of *FAM111B*, thereby mimicking the effects observed with both investigated variants. Encouragingly, pathway enrichment analysis revealed notable similarities between the proteomic profiles of POIKTMP patient-derived cells and those with *FAM111B* knockdown.

One of the pathways impacted by *FAM111B* variants was the UPS (Fig. 7a and Table 2). The causal link between FAM111B decreased expression and UPS alterations became even more conspicuous in *FAM111B*-silenced cells (Fig. 7b), where a greater number of these components exhibited differential expression (Table 2). More importantly, these proteomic changes were echoed by a substantial increase in the accumulation of ubiquitin-modified proteins and alterations in proteasome composition in cells from POIKTMP individuals. However, the underlying cause of protein homoeostasis disruption in cells harbouring *FAM111B* variants remains uncertain, as it is unclear whether this disruption stemmed from heightened ubiquitin conjugation or the observed proteasome reconfiguration (Fig. 9b and c) or even decreased de-ubiquitinating activity.

While the precise molecular mechanisms by which *FAM111B* D-box missense variants regulate the UPS remain elusive, our SPR-MS analysis suggested that they did not involve direct physical binding of variant FAM111B to core UPS components and more specifically to the proteasome. Nevertheless, our investigations indicated an enhanced direct binding to EPB41L3 (also referred to as Band 4.1-like protein 3), which, itself, is predicted to associate with ubiquitin ligases CUL7 and UBE3A, as well as the DNA-binding protein CDC5L (Fig. 8a). One can therefore not exclude that *FAM111B* missense variants of the D-box domain might influence CUL7 and UBE3A through direct interaction with EPB41L3. Among EPB41L3 interaction partners, UBE3A (also referred to as E6AP) stands out due to its ability to bind the proteasome subunit PSMD4.^59^^,^^60^ Importantly, we confirmed an interaction between FAM111B and proteasome subunits through co-precipitation experiments (Fig. 8b), providing further evidence of a connection between these components. Our data also showed that FAM111B co-precipitated with K48-linked ubiquitin-modified proteins in HeLa cells (Fig. 8b) which could potentially include CUL7 substrates. However, whether these interactions are mediated by EPB41L3, UBE3A and/or CUL7 remains an open question and requires additional studies.

Interestingly, EPB41L3 and FAM111B proteins play contrasting roles in tumorigenesis with EPB41L3 acting as a tumour suppressor,^61^ while FAM111B functioning as a protooncogene,^17^^,^^18^^,^^62^ although recent research has challenged this view.^19^ Whether POIKTMP-associated *FAM111B* missense variants preserve the tumorigenic properties of FAM111B remains uncertain, although the observed predisposition to cancer in POIKTMP individuals^31^ lends credibility to this hypothesis. Here, we speculate that FAM111B uses both EPB41L3 and UPS actors to regulate cell proliferation and differentiation, through the formation of a high molecular weight protein complex partly composed of mutant FAM111B, EPB41L3 and CUL7 (Fig. 8c). Indeed, CUL7 is known as a pivotal controller of cell cycle progression, orchestrating the degradation of cyclin D1,^63^^,^^64^ and retaining p53 within the cytosol.^65^ Based on these data, an EPB41L3-CUL7 axis promoted by *FAM111B* missense variants could potentially elucidate the susceptibility to cancer observed in individuals with POIKTMP. These findings, however, need to be validated in additional models beyond patient-derived fibroblasts, ideally in animal systems. A major limitation is the absence of a true orthologue of the human *FAM111B* gene in mice, which complicates the development of conventional knock-in models. Nonetheless, assuming that the interaction between FAM111B and its molecular partners is conserved across species, an alternative approach could involve introducing human FAM111B pathogenic variants into animal models through gene editing or viral-mediated gene delivery, an approach that warrants exploration in future studies.

Our work expanded the cellular phenotype of POIKTMP by introducing protein homoeostasis disruption to its repertoire. In addition, *FAM111B* variants were associated with the acquisition of specific type I IFN gene (Fig. 10 and Supplementary Fig. S7) and proteomic (Table 2) signatures in POIKTMP cells, a well-known autoinflammatory feature of disorders caused by protein homoeostasis perturbations.^47^^,^^48^^,^^66^ Although we do not fully understand the biological relevance of these responses in this context, the fact that IFN induces immunoproteasomes and proteasome activators^67^, ^68^, ^69^ suggested that they may be part of a compensatory mechanism to enhance proteasome function.

Most importantly, our data indicated that the cellular phenotype of POIKTMP shared features with neurological disorders involving protein homoeostasis perturbations. These disorders notably include late-onset neurodegenerative disorders such as Parkinson disease and Alzheimer disease,^70^^,^^71^ as well as the more recently described early-onset neurodevelopmental proteasomopathies caused by loss-of-function variants in proteasomal genes.^47^^,^^72^^,^^73^ This concept might initially pique curiosity, especially when considering that POIKTMP patients typically do not exhibit neurological manifestations.^30^ However, this contrast can be explained by the low expression of *FAM111B* in the brain compared to higher expression in other organs such as the skin, pancreas, and gastrointestinal tract.^74^ One should emphasize that disruptions in protein homoeostasis are also a hallmark of specific autoinflammatory disorders, particularly chronic atypical neutrophilic dermatosis with lipodystrophy and elevated temperature (CANDLE), also known as proteasome-associated autoinflammatory syndromes [PRAAS]. Individuals with CANDLE exhibit skin lesions similar to those observed in POIKTMP.^75^, ^76^, ^77^ This raises the question of whether therapeutic approaches used for CANDLE/PRAAS, such as JAK inhibitors for managing dermatological symptoms,^78^ could potentially be extended to POIKTMP.

More broadly, the involvement of the UPS as a direct contributor to the molecular pathogenesis of POIKTMP opens new theoretical therapeutic avenues, including strategies aimed at restoring proteasome function. Although the efficacy of proteasome and immunoproteasome inhibitors has been extensively characterized, particularly in the treatment of cancer and autoimmune diseases,^79^^,^^80^ the development of proteasome activators remains less advanced. Small molecules, such as the USP14 inhibitor IU1,^81^ p38 MAPK inhibitors,^82^ and phosphodiesterase (PDE) inhibitors,^83^ have demonstrated some capacity to enhance proteasome activity; however, their true clinical relevance has yet to be firmly established. Future studies should consider including POIKTMP models in the preclinical evaluation of these compounds as potential proteasome activators.

In summary, this study improves our knowledge of the clinical history of POIKTMP, which is essential for the development of clinical trials, and on genotype-phenotype correlation, with evidence of a pathophysiological mechanism involving the altered D-box domain, probably responsible for greater phenotype severity. It also provides compelling evidence of ubiquitin ligases interacting with FAM111B partners, potentially involved in UPS dysregulation. The involvement of UPS actors, in both the cellular and molecular mechanisms, seems to be implicated in underpinning POIKTMP. These findings not only advance our understanding of disease pathogenesis but also unveil an unforeseen function of FAM111B in maintaining protein homoeostasis and open up possible therapeutic avenues. Further research is warranted to delve deeper into this function at the molecular level.

## Contributors

V.V., M.M., A.Gu., E.C., B.G., S.Be., S.Ba. and S.M. designed research studies. V.V., M.M., T.B., M.B., A.Gu., E.C., E.D., L.F. K.S., G.M. and L.R. conducted experiments. V.V., M.M., T.B., M.B., A.G., E.C., E.D., A.Gu., J.P., R.R., S.H.R. and S.M. acquired data. V.V., M.M., S.K., T.B., M.B., A.Gu., E.C., E.D., W.D., S. Ba., F.E. and S.M. analysed data. A.B., A.Go., J.P. and R.R. provided reagents. V.V., M.M, S.K., F.E. and S.M. wrote the manuscript. V.V., M.M., F.E. and S.M. verified the underlying data of this manuscript. All authors read and approved the final version of the manuscript.

## Data sharing statement

The mass spectrometry proteomics data have been deposited to the ProteomeXchange Consortium via the PRIDE partner repository. The project accession is PXD046398, project DOI 10.6019/PXD046398. All remaining raw data can be provided upon request to the authors.

## Declaration of interests

The authors declare no conflict of interest.

## Appendix A Supplementary data

Supplementary Figs. S1–S4

REVISION_2_EBioMedicine_Uncropped_Gels
