# Supplementary material for: Ubiquitin-proteasome system dysregulation in FAM111B-related poikiloderma and phenotypic spectrum expansion: new case reports and long-term follow-up
Source: eBioMedicine. 2025 Aug 20;119:105864. doi: 10.1016/j.ebiom.2025.105864 (PMC12396287; doi:10.1016/j.ebiom.2025.105864)
Supplement: Supplementary Figs. S1–S4 [file mmc5.docx]

**Ubiquitin-proteasome system dysregulation in FAM111B-related poikiloderma and phenotypic spectrum expansion: new case reports and long-term follow-up**

Virginie Vignard, Mike Maillasson, Anne Bigot, Sébastien Küry, Thomas Besnard, Martin Broly, Aurélie Guého, Emmanuelle Com, Erica Davis, Wallid Deb, Laëtitia Florenceau, Karen Sobriel, Grégoire Ménard, Betty Gardie, Alice Goldenberg, Joseph Porrmann, Randal Richardson, Léa Ruffier, Smail Hadj-Rabia, Stéphane Bézieau, Sébastien Barbarot, Frédéric Ebstein, Sandra Mercier.

**Supplemental Figures**

Fig. S1. Proteomic analysis of healthy fibroblasts versus POIKTMP-derived patient fibroblasts carrying *FAM111B* variants

Fig. S2. Proteomic analysis comparing healthy fibroblasts to FAM111B-silenced fibroblasts

Fig. S3. Interaction network depicting the association of the common DAPs found between control and both FAM111B-depleted and FAM111B mutant cells

Fig. S4. Analysis of proteasome chymotrypsin-like activity from control and FAM111B mutant fibroblasts.

Fig. S5. Western-blot analysis of the expression of proteasome components in control and POIKTMP patient cells

Fig. S6. Assessment of the type I interferon (IFN) gene expression status in control and FAM111B mutant T cells

**Supplemental Tables**

Table S1. List of antibodies, cell lines and reagents used in this study

Table S2. Detailed genetic and clinical characteristics of POIKTMP patients with FAM111B variants, categorized by cluster localization

Table S3. Summary of clinical features of POIKTMP patients with FAM111B variants, grouped by sex

Table S4. Summary of clinical features of POIKTMP patients with FAM111B variants, categorized by variant clusters

**References**


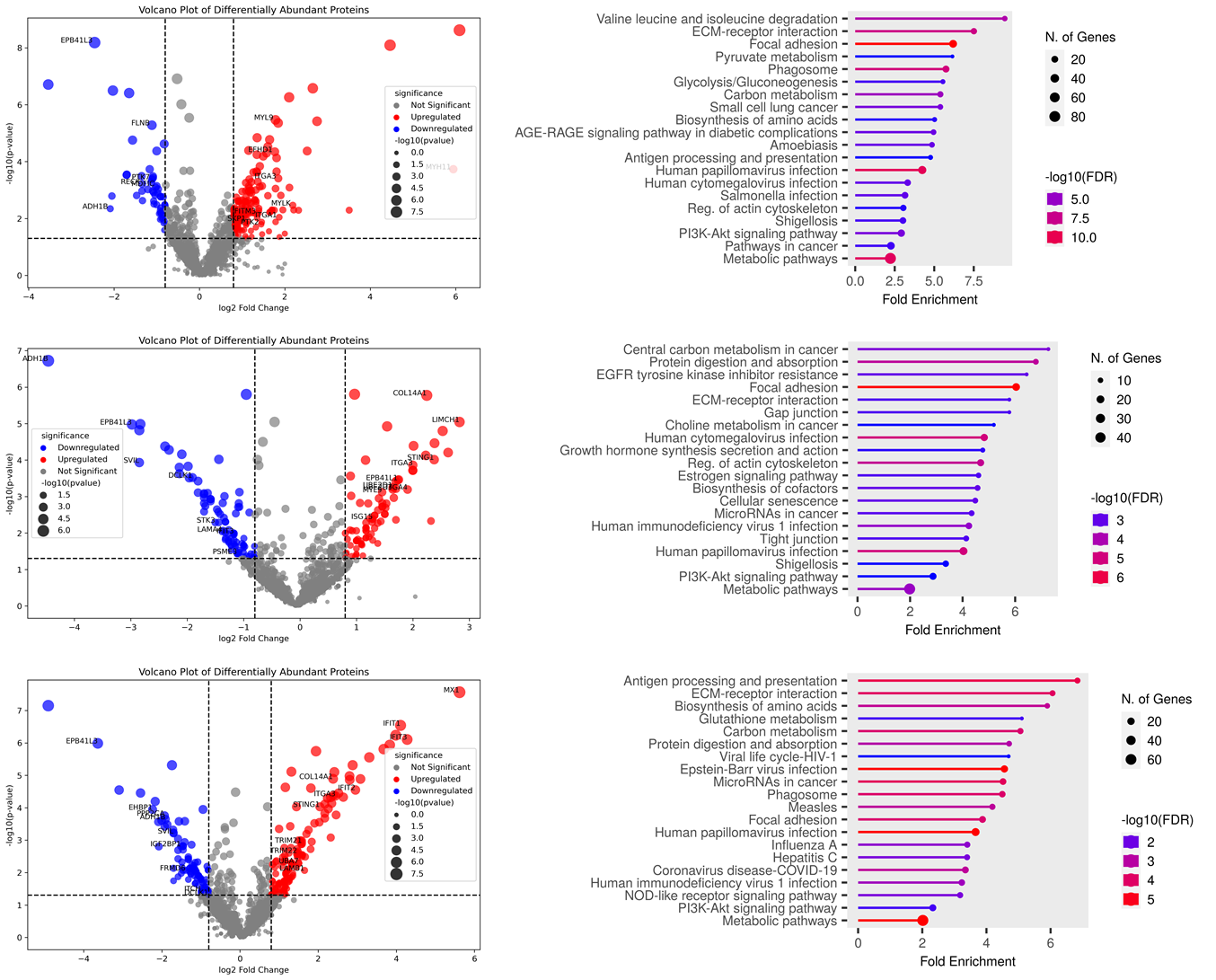


**Fig. S1. Proteomic analysis of healthy fibroblasts versus POIKTMP-derived patient fibroblasts carrying *FAM111B* variants.** Volcano plots illustrating the fold change in protein expression, with upregulated DAPs shown in red and downregulated DAPs in blue in FAM111B-mutant cells compared to age- and sex-matched fibroblasts from healthy individuals. Right panel: Classification of DAPs into KEGG pathways. Data are presented from three independent experiments, shown from top to bottom.


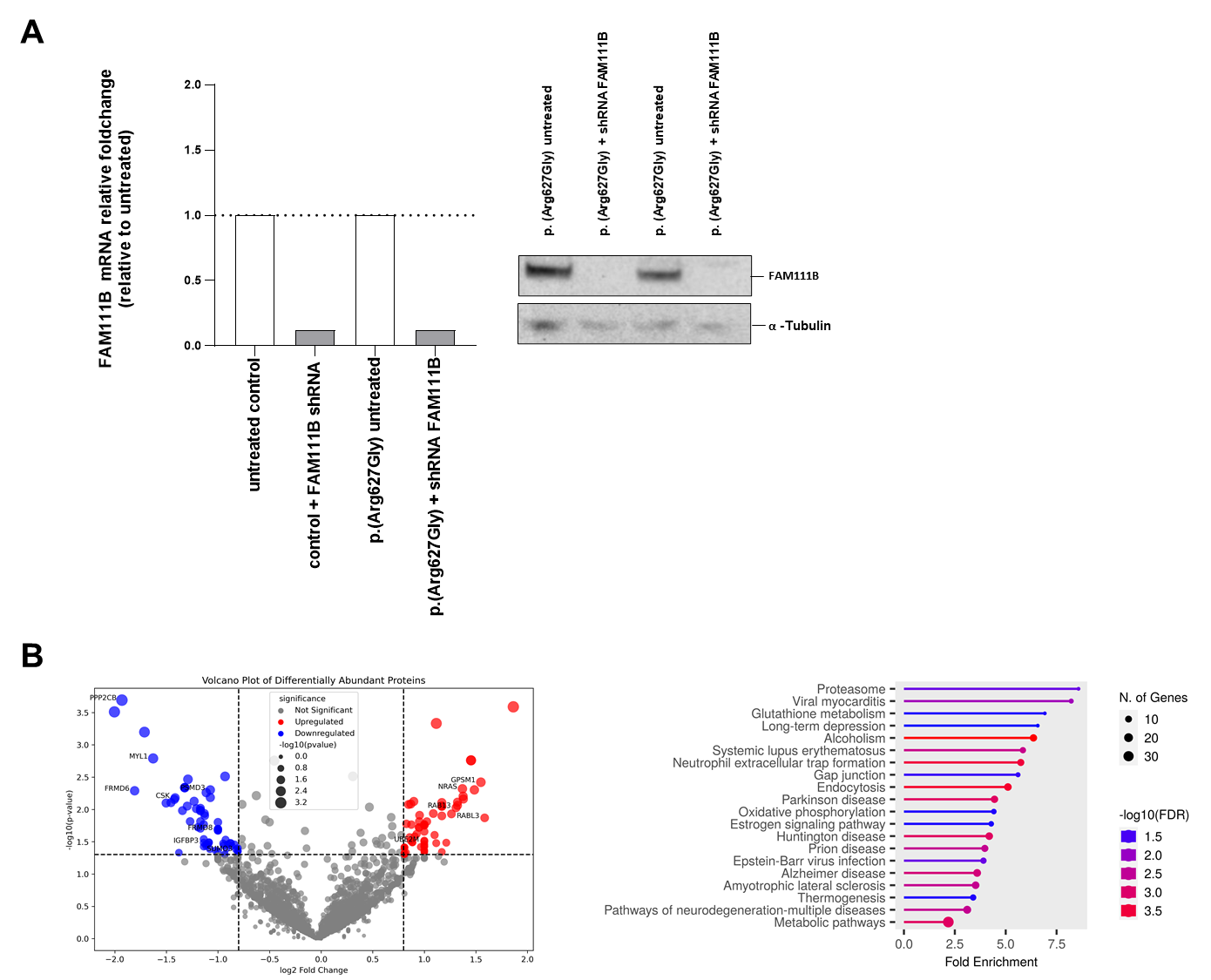


**Fig. S2. Proteomic analysis comparing healthy fibroblasts to *FAM111B*-silenced fibroblasts.** *A*. Left panel, RT-qPCR analysis of FAM111B mRNA from total RNA extracted from untreated fibroblasts or fibroblasts subjected to a 48-hour transfection with either 100 nM of control or FAM111B-specific siRNA. Expression levels were normalized to GAPDH, and relative quantifications (RQ) are presented as fold change over untreated cells. Right panel: Western blot analysis of FAM111B and α-tubulin (loading control) expression in RIPA cell lysates obtained from untreated fibroblasts or fibroblasts exposed to either control or FAM111B siRNA, as described above. *B*. Volcano plots illustrating the fold change in protein expression, with upregulated DAPs shown in red and downregulated DAPs in blue in FAM111B-depleted cells compared to age- and sex-matched fibroblasts from healthy individuals. Shown is also the corresponding classification of DAPs into KEGG pathways.


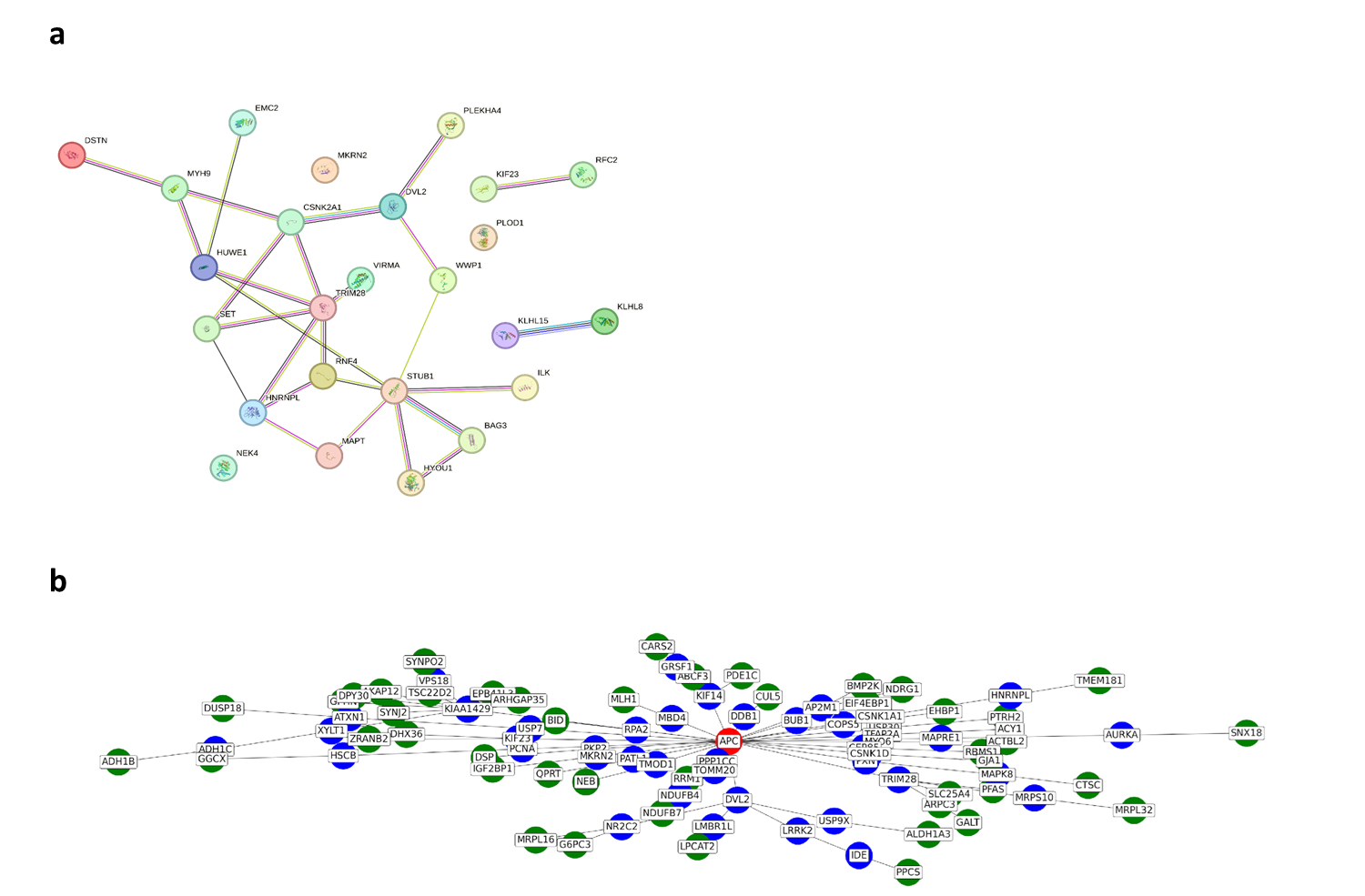


**Fig. S3. Interaction network depicting the association of the common DAPs found between control and both *FAM111B*-depleted and *FAM111B* mutant cells.** *A*. STRING protein-protein interaction network of FAM111B variants. The nodes represent proteins, while the edges signify the quality and quantity of interactions. The intensity of color in the edges denotes the confidence level of a functional association. The node degree indicates the average number of interactions per node and the clustering coefficient specifies the average node density within the map. *B*. Interaction network depicting the association of the nearest neighbor protein (APC/C) and the common DAPs found between control and both FAM111B-depleted and FAM111B mutant cells. The interaction graph was constructed utilizing a unified tool that amalgamates node embedding, deep walk techniques, and the nearest neighbor algorithm. In this visual representation, the central protein is depicted by a red node, determined by the minimum score derived from the nearest neighbor algorithm. Intermediate proteins are denoted by blue nodes, while DAPs or prospective interactors are indicated by green nodes.


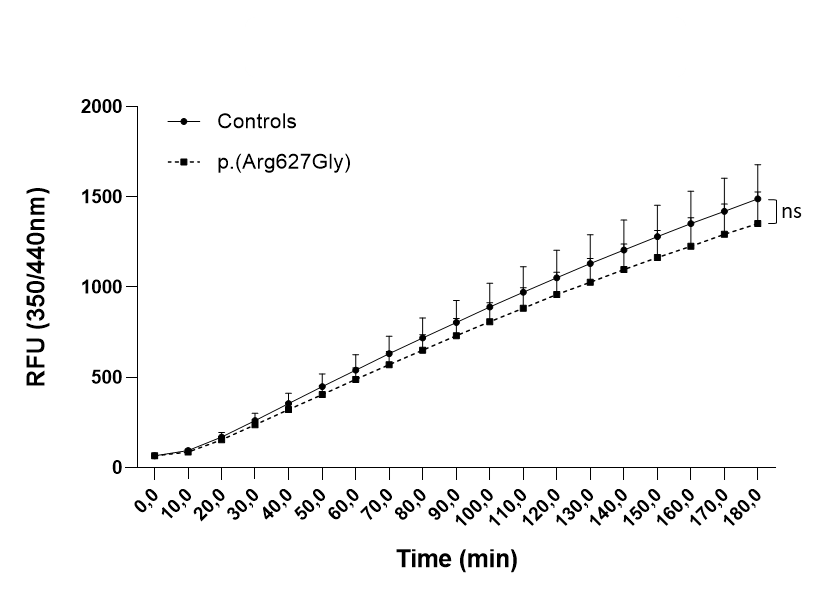


**Fig. S4. Analysis of proteasome chymotrypsin-like activity from control and *FAM111B* mutant fibroblasts.** Healthy donor-derived fibroblasts and fibroblasts isolated from one POIKTMP patient with p.(Arg627Gly) *FAM111B* variant were assessed for their proteasome chymotrypsin-like activity by incubating ten micrograms of TSDG lysates in quadruplicates with 0.1 mM Suc-LLVY in a final volume of 100 μL on 96-well plates. The fluorescence intensity resulting from AMC release was recorded at 360 nm intervals, initially every 10 minutes over a 3-h period of time, as indicated.


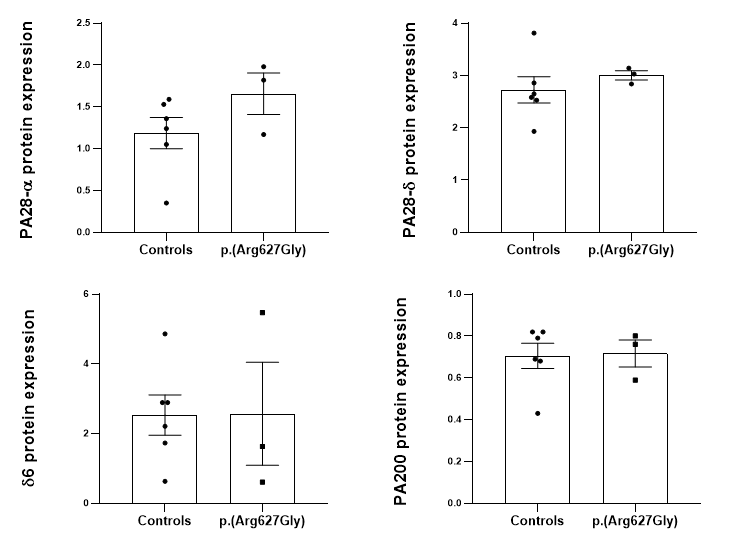


**Fig. S5. Western-blot analysis of the expression of proteasome components in control and POIKTMP patient cells.** RIPA protein lysates from control and POIKTMP patient-derived fibroblasts carrying a p.(Arg627Gly) *FAM111B* variant were separated by SDS-PAGE and subsequently analyzed by western-blotting using antibodies specific for PA28α, PA28γ, α6, PA200, and β-actin (loading control). Immunoreactive bands were quantified by densitometry and the values obtained for PA28α, PA28γ, α6, PA200 were normalized to those of β-actin from the same experiment. Data are presented as mean (±SEM) of PA28α, PA28γ, α6, PA200 to β-actin protein ratios from at least three independent experiments for the control and patient groups.


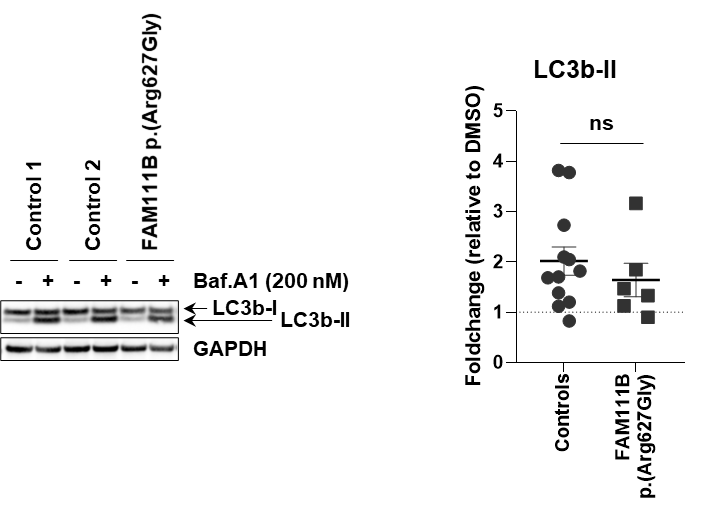


**Fig. S6. Autophagic flux analysis in healthy and POIKTMP patient-derived fibroblasts.** Protein lysates from age- and sex-matched control fibroblasts (controls 1 and 2) were compared to fibroblasts carrying the *FAM111B* p.(Arg627) variant. LC3B-I and LC3B-II levels were analyzed by SDS-PAGE and western blotting following a 12-hour treatment with bafilomycin A1 (Baf.A1) or vehicle (DMSO). Specific antibodies for LC3B and GAPDH (loading control) were used as indicated. The right panel shows a densitometric analysis of LC3B-II immunoreactive bands normalized to GAPDH and expressed relative to vehicle-treated cells. Data are presented as mean values ± SEM from at least six independent experiments.


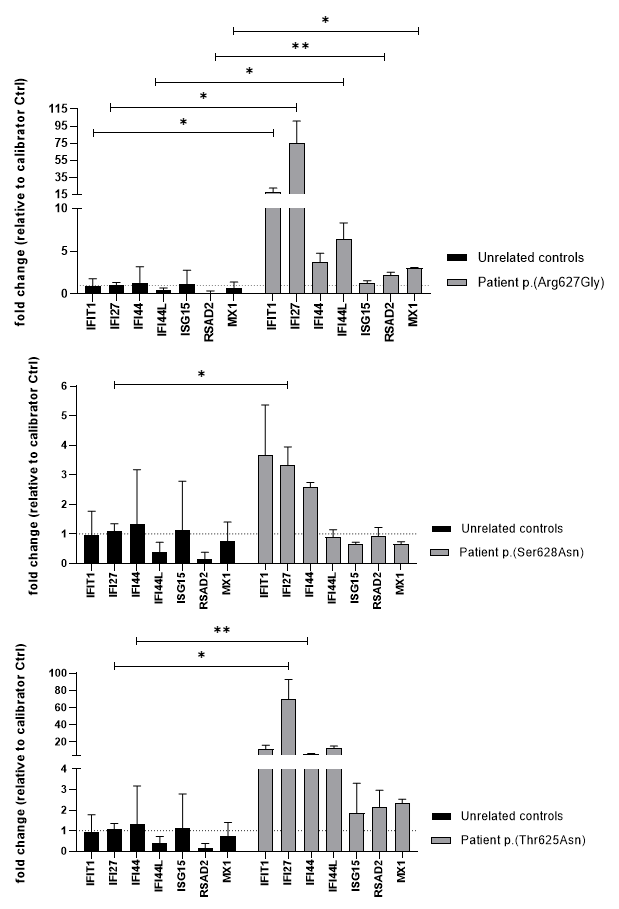


**Fig. S7. Assessment of the type I interferon (IFN) gene expression status in control and FAM111B mutant T cells.** T cells derived from healthy donors and POIKTMP patients with a p.(Arg627Gly), p.(Ser628Asn) or p.(Thr625Asn) *FAM111B* variant were subjected to total RNA extraction for subsequent gene expression analysis of six type I IFN-stimulated genes (ISG) including *IFIT1*, *IFI27*, *IFI44*, *IFI44L*, *ISG15*, and *MX1* by RT-qPCR, as indicated. Expression levels were normalized to *GAPDH* and *HPRT1* and presented as ISG fold change in POIKTMP patient over one healthy donor carrying wild-type FAM111B which was used as calibrator control for all analyzed samples. Statistical significance was assessed by paired Student t test (**p*<0.05, ***p*<0.01).

**Table S1. List of antibodies used in this study**

See Excel file.

**Table S2. Detailed genetic and clinical characteristics of POIKTMP patients with FAM111B variants, categorized by cluster localization**

See Excel file.

**Table S3. Summary of clinical features of POIKTMP patients with FAM111B variants, grouped by sex**

See Excel file.

**Table S4. Summary of clinical features of POIKTMP patients with FAM111B variants, categorized by variant clusters**

See Excel file.

**References**

1. Thiebaut AM, et al. Thrombolysis by PLAT/tPA increases serum free IGF1 leading to a decrease of deleterious autophagy following brain ischemia. *Autophagy*. 2022;18(6):1297–1317.

2. Le Faouder J, et al. Fish Hydrolysate Supplementation Prevents Stress-Induced Dysregulation of Hippocampal Proteins Relative to Mitochondrial Metabolism and the Neuronal Network in Mice. *Foods*. 2022;11(11). https://doi.org/10.3390/foods11111591.

3. Girard O, et al. Naive Pluripotent and Trophoblastic Stem Cell Lines as a Model for Detecting Missing Proteins in the Context of the Chromosome-Centric Human Proteome Project. *J Proteome Res*. 2023;22(4):1148–1158.

4. Bouyssié D, et al. Proline: an efficient and user-friendly software suite for large-scale proteomics. *Bioinformatics*. 2020;36(10):3148–3155.

5. Méar L, et al. The Eutopic Endometrium Proteome in Endometriosis Reveals Candidate Markers and Molecular Mechanisms of Physiopathology. *Diagnostics (Basel)*. 2022;12(2). https://doi.org/10.3390/diagnostics12020419.

6. Fermin D, et al. Abacus: a computational tool for extracting and pre-processing spectral count data for label-free quantitative proteomic analysis. *Proteomics*. 2011;11(7):1340–5.

7. Pham T V, et al. On the beta-binomial model for analysis of spectral count data in label-free tandem mass spectrometry-based proteomics. *Bioinformatics*. 2010;26(3):363–9.

8. Zhou H, et al. Comprehensive analysis of prognostic value, immune implication and biological function of CPNE1 in clear cell renal cell carcinoma. *Front Cell Dev Biol*. 2023;11:1157269.

9. Liebermeister W, et al. Visual account of protein investment in cellular functions. *Proc Natl Acad Sci U S A*. 2014;111(23):8488–93.

10. Szklarczyk D, et al. STRING v10: protein-protein interaction networks, integrated over the tree of life. *Nucleic Acids Res*. 2015;43(Database issue):D447-52.
