# Supplementary material for: Ubiquitin-proteasome system dysregulation in FAM111B-related poikiloderma and phenotypic spectrum expansion: new case reports and long-term follow-up
Source: eBioMedicine. 2025 Aug 20;119:105864. doi: 10.1016/j.ebiom.2025.105864 (PMC12396287; doi:10.1016/j.ebiom.2025.105864)
Supplement: REVISION_2_EBioMedicine_Uncropped_Gels [file mmc6.pdf]

4a

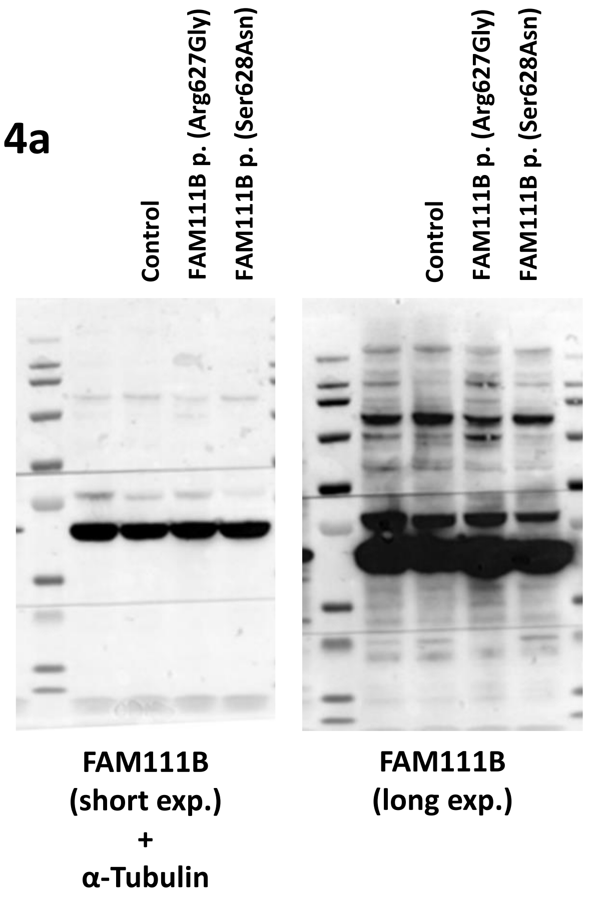
Control
FAM111B p. (Arg627Gly)
FAM111B p. (Ser628Asn)
Control
FAM111B p. (Arg627Gly)
FAM111B p. (Ser628Asn)
FAM111B
(short exp.)
+
α-Tubulin
FAM111B
(long exp.)

4c

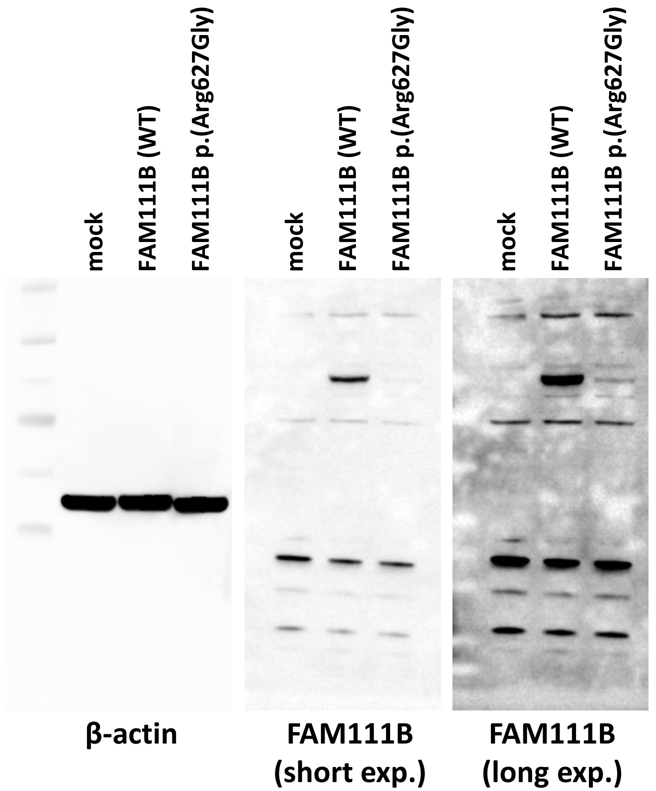
mock
FAM111B (WT)
FAM111B p.(Arg627Gly)
mock
FAM111B (WT)
FAM111B p.(Arg627Gly)
mock
FAM111B (WT)
FAM111B p.(Arg627Gly)
β-actin
FAM111B
(short exp.)
FAM111B
(long exp.)

5a

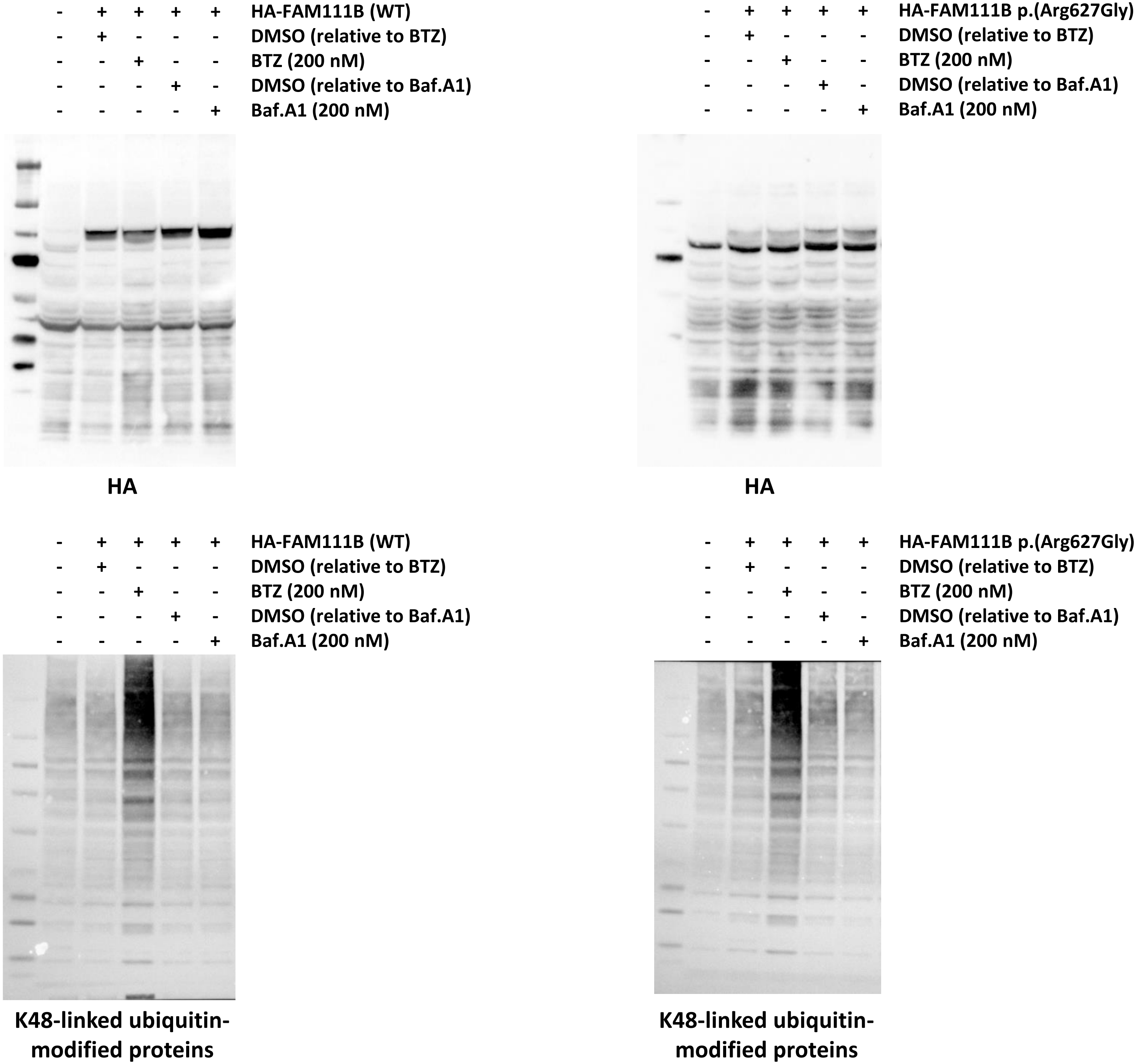
- + + + + HA-FAM111B (WT)
- + - - - DMSO (relative to BTZ)
- - + - - BTZ (200 nM)
- - - + - DMSO (relative to Baf.A1)
- - - - + Baf.A1 (200 nM)
HA
- + + + + HA-FAM111B p.(Arg627Gly)
- + - - - DMSO (relative to BTZ)
- - + - - BTZ (200 nM)
- - - + - DMSO (relative to Baf.A1)
- - - - + Baf.A1 (200 nM)
HA
- + + + + HA-FAM111B (WT)
- + - - - DMSO (relative to BTZ)
- - + - - BTZ (200 nM)
- - - + - DMSO (relative to Baf.A1)
- - - - + Baf.A1 (200 nM)
K48-linked ubiquitin-modified proteins
- + + + + HA-FAM111B p.(Arg627Gly)
- + - - - DMSO (relative to BTZ)
- - + - - BTZ (200 nM)
- - - + - DMSO (relative to Baf.A1)
- - - - + Baf.A1 (200 nM)
K48-linked ubiquitin-modified proteins

5a

-	+	+	+	+	HA-FAM111B (WT)
-	+	-	-	-	DMSO (relative to BTZ)
-	-	+	-	-	BTZ (200 nM)
-	-	-	+	-	DMSO (relative to Baf.A1)
-	-	-	-	+	Baf.A1 (200 nM)

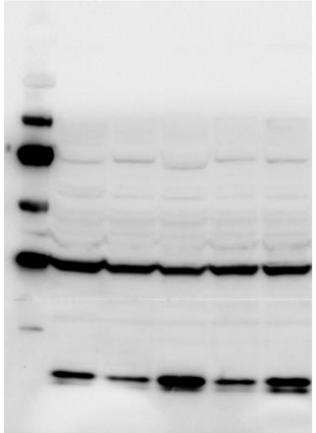

GAPDH
+
LC3B

-	+	+	+	+	HA-FAM111B p.(Arg627Gly)
-	+	-	-	-	DMSO (relative to BTZ)
-	-	+	-	-	BTZ (200 nM)
-	-	-	+	-	DMSO (relative to Baf.A1)
-	-	-	-	+	Baf.A1 (200 nM)

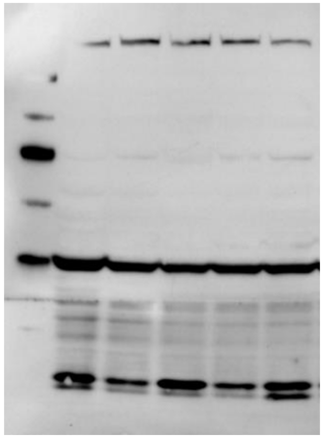

GAPDH
+
LC3B

5c

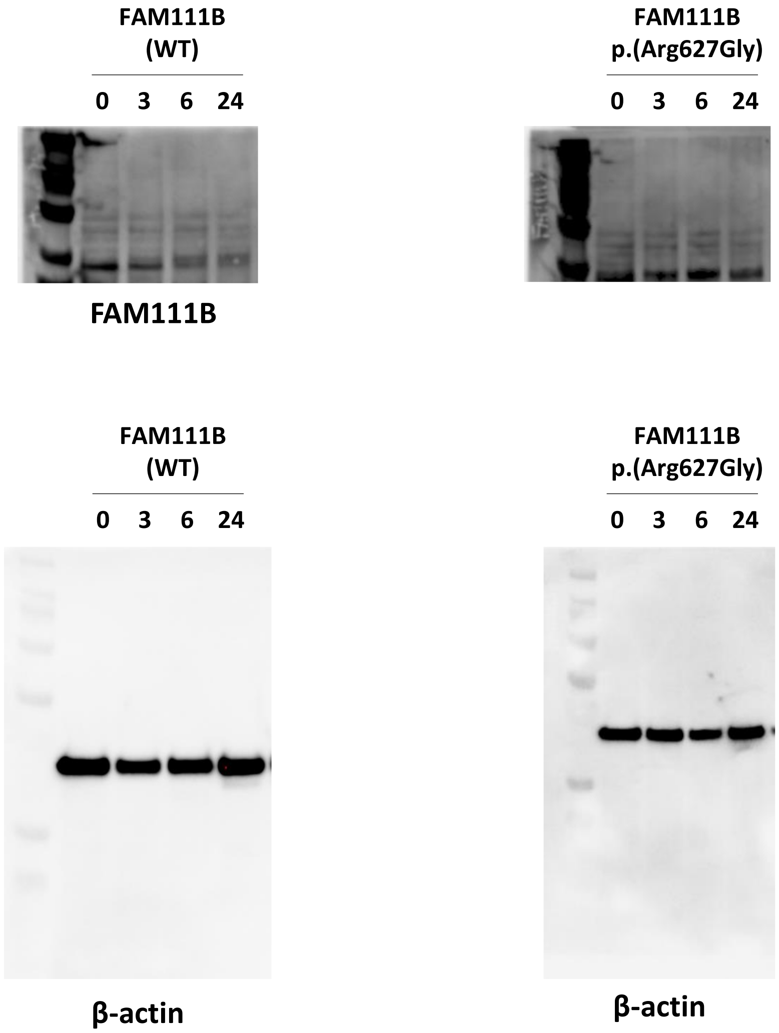
FAM111B (WT)
0 3 6 24
FAM111B p.(Arg627Gly)
0 3 6 24
FAM111B
FAM111B (WT)
0 3 6 24
FAM111B p.(Arg627Gly)
0 3 6 24
β-actin
β-actin

8b

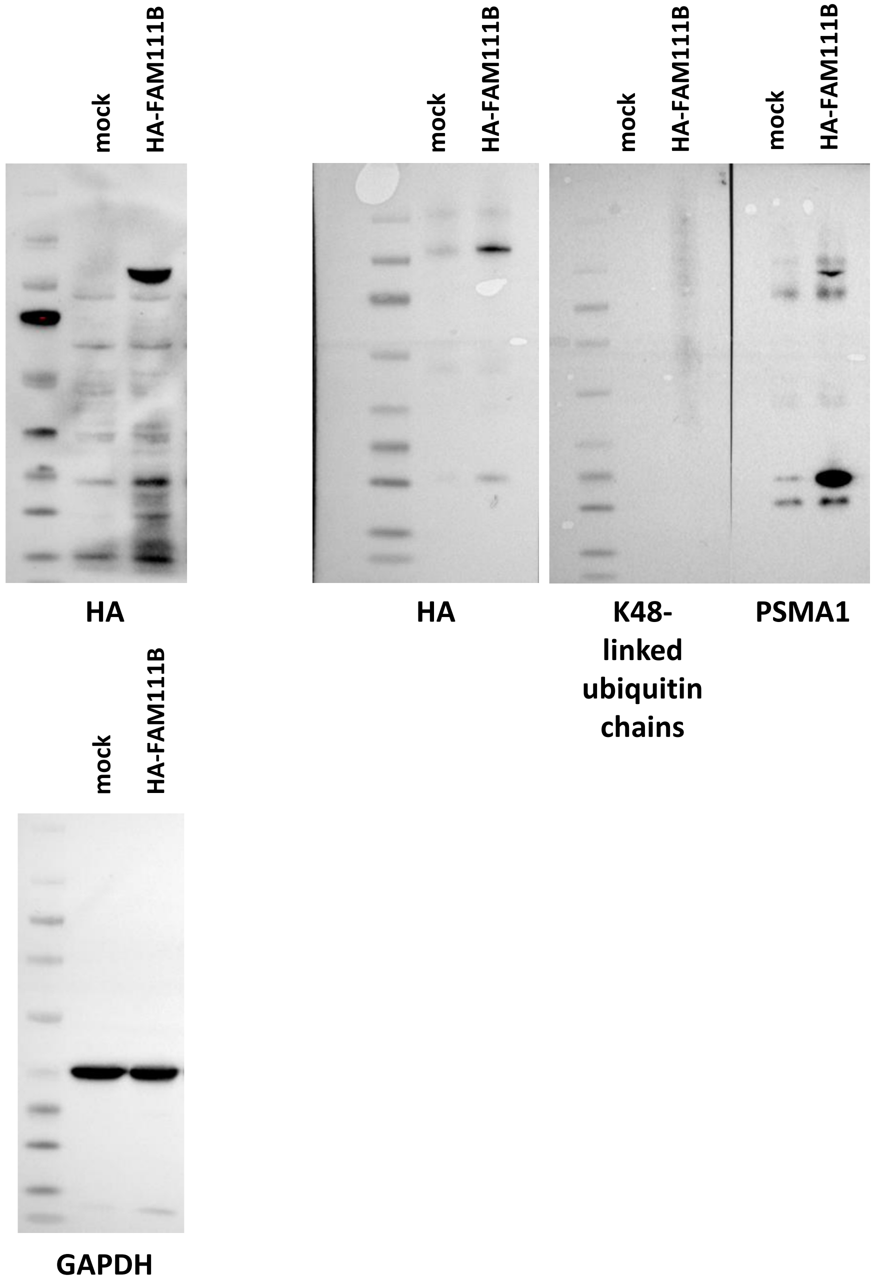
mock
HA-FAM111B
HA
mock
HA-FAM111B
mock
HA-FAM111B
mock
HA-FAM111B
HA
K48-linked ubiquitin chains
PSMA1
mock
HA-FAM111B
GAPDH

8c

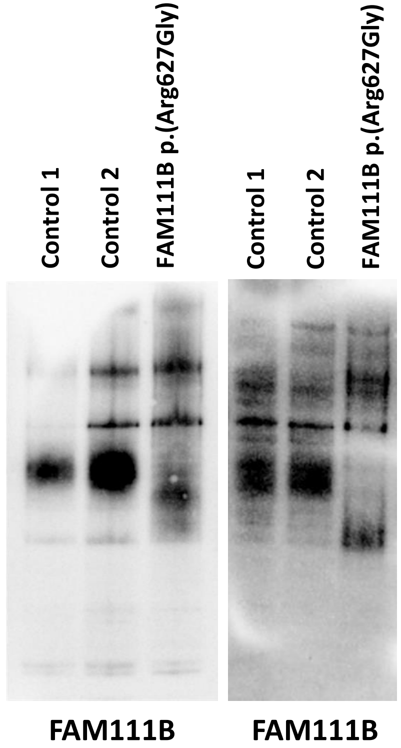
Control 1
Control 2
FAM111B p.(Arg627Gly)
Control 1
Control 2
FAM111B p.(Arg627Gly)
FAM111B
FAM111B

9a

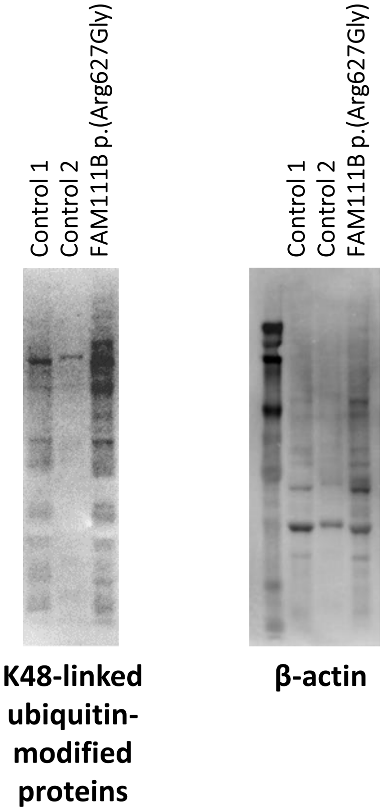
Control 1
Control 2
FAM111B p.(Arg627Gly)
Control 1
Control 2
FAM111B p.(Arg627Gly)
K48-linked ubiquitin-modified proteins
β-actin

9b

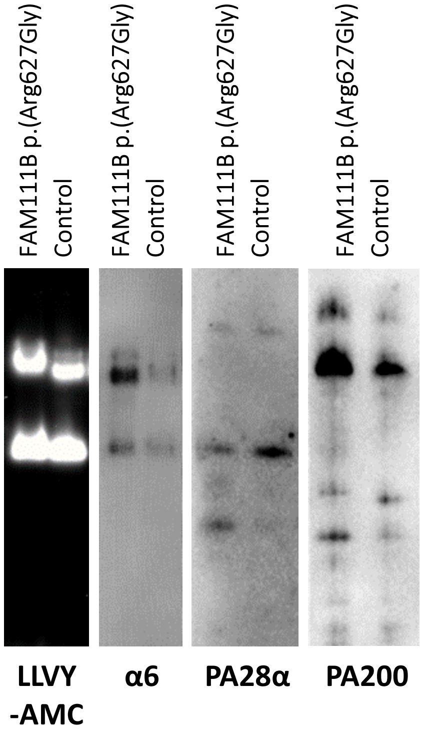
FAM111B p.(Arg627Gly)
Control
FAM111B p.(Arg627Gly)
Control
FAM111B p.(Arg627Gly)
Control
FAM111B p.(Arg627Gly)
Control
LLVY -AMC
α6
PA28α
PA200
